# Cryo-EM structures of LolCDE reveal the molecular mechanism of bacterial lipoprotein sorting in *Escherichia coli*

**DOI:** 10.1371/journal.pbio.3001823

**Published:** 2022-10-13

**Authors:** Weiwei Bei, Qingshan Luo, Huigang Shi, Haizhen Zhou, Min Zhou, Xinzheng Zhang, Yihua Huang

**Affiliations:** 1 National Laboratory of Biomacromolecules, Institute of Biophysics, Chinese Academy of Sciences, Beijing, China; 2 University of Chinese Academy of Sciences, Beijing, China; 3 Institute of Bio-analytical Chemistry, School of Chemical Engineering, Nanjing University of Science and Technology, No.200 Xiao Ling Wei Street, Nanjing, China; Rutgers University-Robert Wood Johnson Medical School, UNITED STATES

## Abstract

Bacterial lipoproteins perform a diverse array of functions including bacterial envelope biogenesis and microbe–host interactions. Lipoproteins in gram-negative bacteria are sorted to the outer membrane (OM) via the localization of lipoproteins (Lol) export pathway. The ATP-binding cassette (ABC) transporter LolCDE initiates the Lol pathway by selectively extracting and transporting lipoproteins for trafficking. Here, we report cryo-EM structures of LolCDE in apo, lipoprotein-bound, and AMPPNP-bound states at a resolution of 3.5 to 4.2 Å. Structure-based disulfide crosslinking, photo-crosslinking, and functional complementation assay verify the apo-state structure and reveal the molecular details regarding substrate selectivity and substrate entry route. Our studies snapshot 3 functional states of LolCDE in a transport cycle, providing deep insights into the mechanisms that underlie LolCDE-mediated lipoprotein sorting in *E*. *coli*.

## Abstract 2

Lipoproteins in Gram-negative bacteria are sorted to the outer membrane via the Lol export pathway. The ABC transporter LolCDE initiates this pathway by selectively extracting and transporting lipoproteins for trafficking; this study provides a structural basis for the LolCDE-mediated bacterial lipoprotein sorting, with implications for novel antibiotic design against Gram-negative bacterial pathogens.

## Introduction

**Fig 1:**
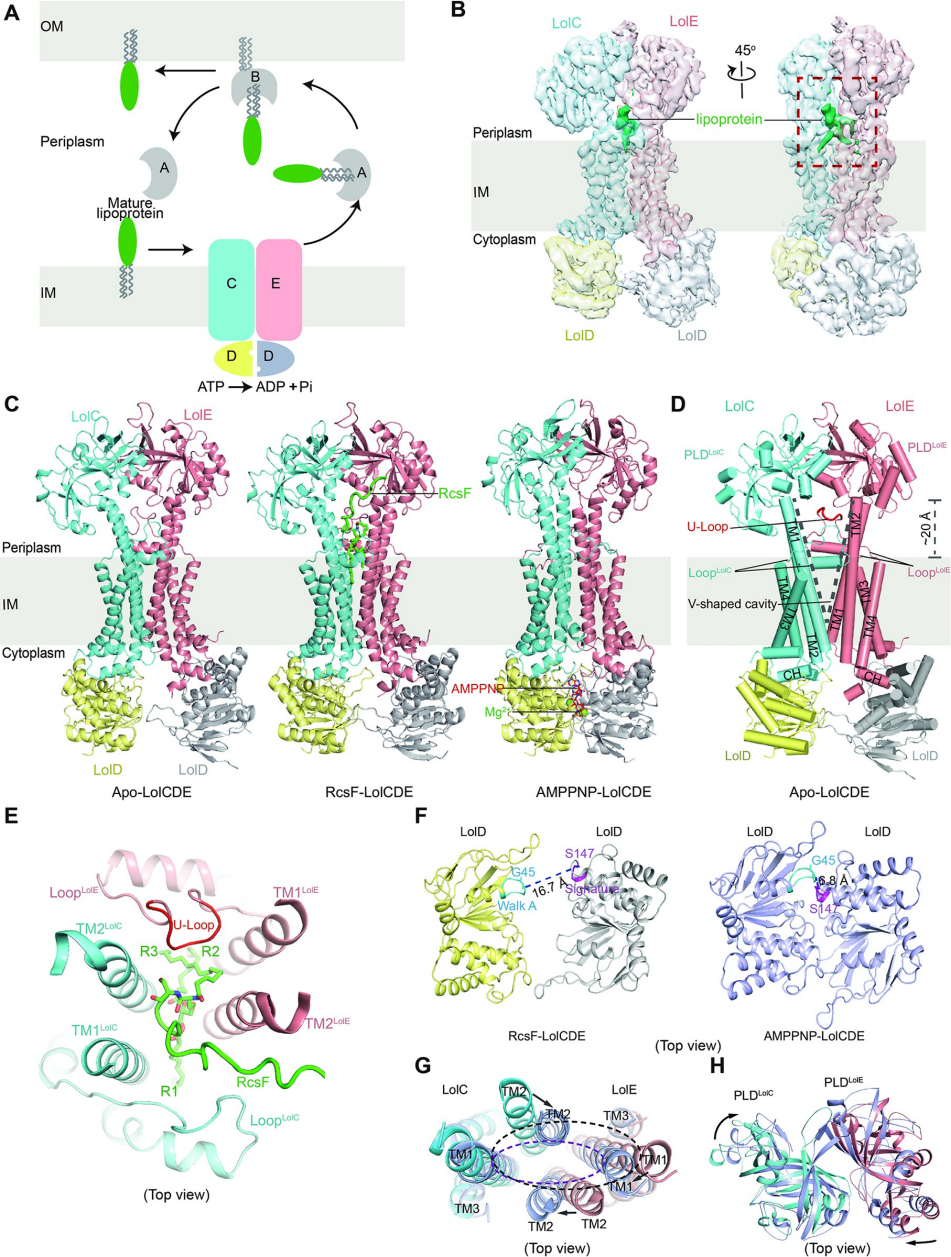
Cryo-EM structures of LolCDE in different states. (**A**) Schematics of the Lol pathway. Letters A to E designate LolA to LolE, respectively. (**B**) The 4.0-Å cryo-EM map of LolCDE. LolC, LolE and 2 copies of LolD are colored in blue, pink, yellow, and grey, respectively. The lipoprotein densities are shown in green. (**C**) Ribbon diagram of apo, RcsF-bound and AMPPNP-bound LolCDE structures. Mg^2+^ is shown in green spheres and AMPPNP in red sticks. (**D**) Cylindrical helix cartoon representation of structure motifs of apo-LolCDE. The U-Loop is highlighted in red. Dashed lines indicate the V-shaped cavity of LolCDE. (**E**) Top view of the V-shaped cavity, configured by TM1s and TM2s of LolC and LolE, Loop^LolC^ and Loop^LolE^. (**F-H**) Conformational changes of LolCDE domains upon AMPPNP binding. (**F**) Top view of NBDs of RcsF-LolCDE (left) and AMPPNP-LolCDE (right). (**G**) Overlay of TMDs of RcsF-LolCDE (blue and pink) and AMPPNP-LolCDE (purple). The arrows indicate direction of TMs moving upon AMPPNP binding. The black and red dashed lines indicate the changes of the V-shaped cavity. (**H**) Overlay of PLDs of RcsF-LolCDE (blue and pink) and AMPPNP-LolCDE (purple). The arrows indicate directions of PLDs rotation upon AMPPNP binding.

The outer membrane (OM), hallmark of gram-negative bacteria, lies at the frontline of interaction with environment serving as a potent permeability barrier that prevents entry of many toxic substances into the cell [1,2]. Central to OM biogenesis and physiology are the lipoproteins that peripherally anchored to the membrane via their N-terminal lipid moiety. OM lipoproteins underpin the functioning of a diverse array of machineries that are responsible for the lipopolysaccharides (LPS) export, assembly of integral OM proteins and peptidoglycan cell wall, to name a few [3–6]. Lipoproteins are therefore indispensable for the survival of gram-negative bacteria [7], bearing important implications for efforts to develop novel antimicrobial agents against multidrug-resistant bacteria [8–12]. In gram-negative bacteria, lipoproteins are synthesized in the cytoplasm and matured in the inner membrane (IM). In *Escherichia coli*, maturation of lipoproteins occurs on the periplasmic face of the IM and involves consecutive modifications by 3 membrane-bound enzymes Lgt, Lsp, and Lnt [13–18]. Thereafter, they are either retained in the IM or transferred to the inner leaflet of the OM, the determinant being the presence of Asp at +2 position followed by certain residues at +3, the so-called Lol avoidance signal [19,20]. Lipoproteins with the signal remain in the IM, whereas others enter the Lol pathway for transport to the OM [21,22]. The Lol pathway comprises 5 Lol proteins, LolA-E [7,20] (Fig 1A). Among them, LolCDE, an IM-embedded ATP-binding cassette (ABC) transporter initiates lipoprotein sorting by selectively extracting OM-destined lipoproteins from the outer leaflet of the IM [23] and transfers them to LolA [24,25], a periplasmic chaperone. LolB, itself an OM-localized lipoprotein, accepts LolA-bound lipoproteins in a “mouth-to-mouth” manner and inserts them into the inner leaflet of the OM via their N-terminal acyl chains [26–29]. At least 90 different lipoproteins have been identified as substrates of the Lol pathway in *E*. *coli*, and they perform a diverse array of functions after being sorted to their final destinations [30,31].

Importantly, LolCDE belongs to the ABC3 superfamily of ABC transporters, also known as type VII ABC transporters [32], does not transport substrates across the IM, rather they selectively extract mature lipoproteins from the outer leaflet of the IM and transfers them to LolA, propelled by cytoplasmic ATP hydrolysis. This is in stark contrast to canonical ABC transporter. Recently, Kaplan and colleagues reported the crystal structure of LolA in complex with the periplasmic domain of the LolC [25]. Tang and colleagues and Sharma and colleagues determined cryo-EM structures of LolCDE in different conformational states [33,34]. High-resolution structures of the Lol components promise to greatly advance our understanding of the molecular mechanism of lipoprotein biogenesis. Here, we reported cryo-EM structures of LolCDE in its apo, RcsF- and AMPPNP-bound states. We point out that the apo-LolCDE structure we obtained is strikingly different from what was previously reported [33]. Furthermore, our structure-based functional analysis reveals a clear path through which lipoproteins enter the substrate-binding cavity of LolCDE.

## Results

### Overall structures of apo-LolCDE, RcsF-LolCDE, and AMPPNP-LolCDE in nanodisc

LolCDE proteins were expressed in *E*. *coli* BL21 (DE3), purified in dodecyl maltoside (DDM), and reconstituted in nanodisc (S1A Fig). The LolCDE structure was initially determined at an overall resolution of 4.0 Å (S2A–S2C Fig), yet extra densities that resemble acyl chains of a lipoprotein were observed in the cavity of the transmembrane domains (TMDs) of LolC and LolE (Fig 1B). SDS-PAGE and mass spectrometry analysis identified that endogenous RcsF, a lipoprotein that is involved in the Rcs (regulator of capsule synthesis) system [35,36] was copurified with LolCDE (Figs 1B and S1D and S1E). To reveal the interaction details of RcsF-LolCDE, RcsF was coexpressed with LolCDE to attain a 1:1 stoichiometry for structure determination (S1B and S1D Fig). This allowed us to obtain a 3.5-Å cryo-EM map for RcsF-LolCDE (S3 Fig). Atomic models of the RcsF-LolCDE complex were therefore built with certainty (Figs 1C and S4).

As the initial LolCDE sample contained predominantly apo-LolCDE particles (S1A and S1D Fig), we reclassified these particles using a soft mask to exclude densities with bound RcsF. After several rounds of 3D classification and calculation, we finally determined the apo-LolCDE structure at a 4.2-Å resolution using 135,391 selected particles. Multiple verification runs were taken to eliminate any reference bias in the method (S2C–S2I Fig). Further, to obtain the structure of the ATP-bound state of LolCDE, we preincubated LolCD^E171Q^E, a catalytically dead LolCDE mutant with 2 mM AMPPNP (the nonhydrolysable ATP analogue). Thereby, we obtained a structure of AMPPNP-LolCDE determined at 3.6 Å resolution (S5, S6A and S6B Figs).

The overall structure of apo-LolCDE resembles a dumbbell (Fig 1D). In spite of the fact that LolC and LolE share only 26.0% sequence identity (S7A Fig), they adopt a similar fold. It comprises a TMD domain of 4 transmembrane helices (TM1 to TM4), a periplasmic loop connecting TM3 and TM4 [denoted as Loop^LolC^ (residues: Ser338-Val358) and Loop^LolE^ (residues: Ser342-Ser373), respectively] and a periplasmic localized domain (PLD) stemming from TM1 and TM2 (Fig 1D). Superposition of LolC with LolE gives an RMSD of 2.27 Å for PLDs (over 134 Cα atoms) and 1.63 Å for TMDs (over 176 Cα atoms) (S7B and S7C Fig). The TMD domains of LolC and LolE are assembled primarily via intermolecular TM1–TM2 interactions (Fig 1D). Strikingly, the TM1s and TM2s of both LolC and LolE are bent outwards creating a V-shaped substrate-binding cavity open to the periplasm. Both PLDs are in elevated position about 20 Å above the plane of membrane, as a result of TM1s and TM2s extending into the periplasm (Fig 1D). The feature echoes what was observed in the structures of the toxin and antibiotic ABC transporter MacB [37,38]. Moreover, the 2 PLDs interact closely with each other, manifesting a twist of approximately 20° perpendicular to the membrane plane. The surface burial area is calculated to be 405.7 Å^2^.

Similar to LptB_2_FG, an ABC transporter that extracts LPS from the IM [39–42], the substrate-bound LolCDE adopts almost the same conformation as apo-LolCDE (with an RMSD of 1.28 Å over 1158 aligned Cα atoms) (S8A Fig). It is worth to note that in both apo-LolCDE and RcsF-LolCDE structures, the V-shaped cavity is surrounded by 2 structured motifs, Loop^LolC^ and Loop^LolE^ (Fig 1E). This creates 2 intermolecular interfaces, that is, the Loop^LolC^-TM2^LolE^ and the Loop^LolE^-TM2^LolC^ interfaces, perhaps serving as entry gates for lipoprotein substrates. In the V-shaped cavity, the 3 acyl chains of RcsF straddles the TMD interface of LolC and LolE, with 1 acyl chain, R1, residing on the Loop^LolC^ side, and the other two, R2 and R3, extending to the Loop^LolE^ side (Fig 1E). This is in line with the 2 recently reported cryo-EM structures of the lipoprotein-bound LolCDE complexes [33,34]. Importantly, the proteinaceous part of RcsF extends from the V-shaped cavity and appears only on the Loop^LolC^ side, suggesting that RcsF might enter the cavity laterally via the Loop^LolC^-TM2^LolE^ interface. In addition, a U-shaped loop (denoted as U-Loop) from Loop^LolE^ dips into the V-shaped cavity, presumably buttressing the configuration of the V-shaped cavity (Fig 1E).

In stark contrast to lipoprotein binding, AMPPNP binding causes significant conformational changes of LolCDE (S8B Fig and S1 Movie). First, binding of AMPPNP leads to a tight dimerization of the 2 LolDs, resulting in the Cα-Cα distance between Gly45 of the Walk A motif in 1 LolD and Ser147 of the signature motif in the other shortened from 16.7 Å to 6.8 Å (Fig 1F). Second, the V-shaped cavity is flattened, and its periplasmic surface area is reduced to approximately one-third (Fig 1G). Third, in the periplasm, the 2 PLDs become more compact, with a further increase of surface area of 21.1 Å^2^, in comparison to RcsF-LolCDE (Fig 1H). Consequently, no lipoprotein is found in the AMPPNP-LolCDE structure, and the U-Loop is squeezed out (S8C Fig).

In summary, while our RcsF-LolCDE and AMPPNP-LolCDE structures overlay well with the recently published structures [33,34] (S9A and S9B Fig), our apo-LolCDE structure is strikingly different from the apo-LolCDE structure (denoted as apo-LolCDE*, PDB code: 7ARI) by Tang and colleagues [33] (S9C Fig). This prompted us to further verify the apo-LolCDE structure using biochemical approaches.

### Verification of the apo-LolCDE structure

**Fig 2:**
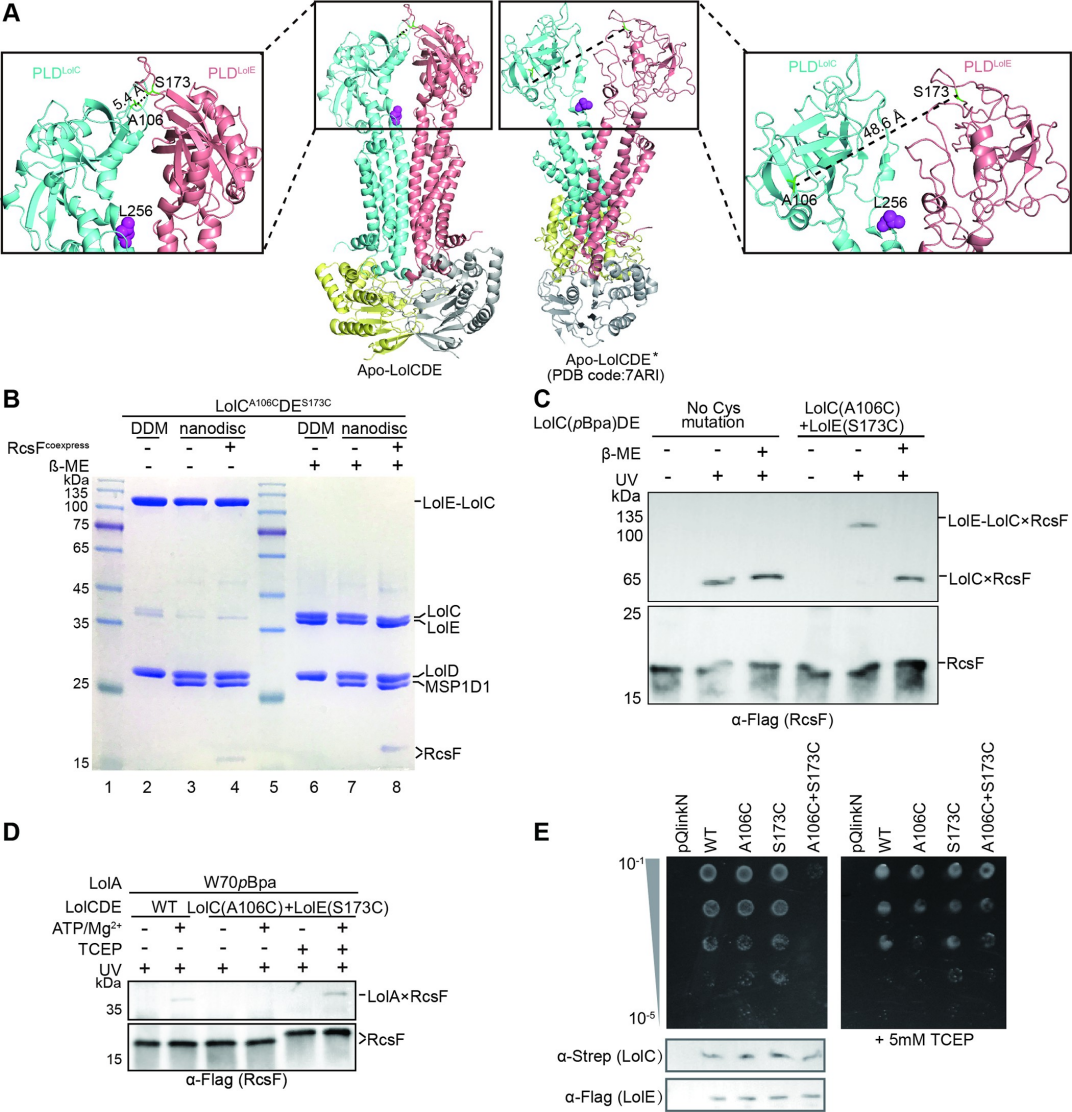
Verification of the apo-LolCDE structure. (**A**) Comparison of our apo-LolCDE structure (left) to the apo-LolCDE* structure (right, PDB code:7ARI). Zoom-in view showing 2 amino acids in 2 PLDs, Ala106 and Ser173, which were replaced with cysteines in (B to E). Leu256^LolC^ shown in purple spheres was substituted with *p*BPA for in vitro photo-crosslinking in (C). (**B**) Coomassie-stained SDS–PAGE gel assessing disulfide bond formation of LolC^A106C^DE^S173C^ and RcsF-LolC^A106C^DE^S173C^. The samples of lanes 2 through 4 were supplemented with SDS loading dye without β-ME, and the samples of lanes 6 through 8 were supplemented with SDS loading dye with β-ME. Note that RcsF migrates slower after addition of reducing agent. (**C**) In vitro photo-crosslinking. LolC^L256*p*BPA^DE proteins with or without 2 cysteine mutations were reconstituted with RcsF in nanodisc. The LolC×RcsF and the LolE-LolC×RcsF adducts were detected by immunoblotting. (**D**) The in vitro lipoprotein transport assays. To break intermolecular disulfide bond, the nanodisc-embedded RcsF-LolC^A106C^DE^S173C^ protein was incubated with TCEP prior to the addition of LolA (W70*p*BPA). (**E**) Complementation assays. The dilutions were spotted on LB plates with (right) or without (left) TCEP. Protein leaky expression levels of the LolC and LolE proteins were detected by western blotting (bottom). Data shown in (B to E) are representatives of 3 replicates.

In our apo-LolCDE structure, the Cα-Cα distance between Ala106 in PLD^LolC^ and Ser173 in PLD^LolE^ is only 5.4 Å, whereas the distance is increased to 48.6 Å in apo-LolCDE* [33] (Fig 2A). To validate our structure, 2 cysteines were incorporated into LolCDE to generate LolC^A106C^DE^S173C^ mutant in order to probe the distance between LolC and LoE in LolCDE. In the absence of reducing reagents, we observed intermolecular disulfide bond between LolC^A106C^ and LolE^S173C^ almost fully formed in the purified LolC^A106C^DE^S173C^ protein. Addition of β-mercaptoethanol (β-ME) disrupted the intermolecular disulfide bond (Fig 2B). We thus conclude that the arrangement of the 2 PLDs in LolCDE agrees well with our apo-LolCDE structure. Furthermore, the crosslinks remained the same, regardless of the presence of RcsF (Fig 2B). These results further confirmed that both the apo- and RcsF-bound LolCDE adopt the same conformations as we observed.

To investigate whether conformational changes of apo-LolCDE are required for RcsF entry into the V-shaped cavity, we incorporated a photo-crosslinkable unnatural amino acid (*p*-benzoyl-phenylalanine (*p*BPA)) [43] at Leu256 of LolC (Fig 2A), whose side chain points to the V-shaped cavity. As shown in Fig 2C, LolC×RcsF adducts are detected when both RcsF and LolCDE were reconstituted in nanodisc, demonstrating that the exogenously added RcsF is able to enter the V-shaped cavity of LolC^L256*p*BPA^DE (no Cys mutations). Furthermore, we also detected the LolE-LolC×RcsF adducts in LolC^A106C^DE^S173C^ sample upon UV radiation. This observation implicates that conformational change of the 2 PLDs of LolC and LolE is not required for the entry of RcsF into the V-shaped cavity (Fig 2C). We observed, however, post-RcsF entry into the V-shaped cavity of disulfide-bonded LolC^A106C^DE^S173C^ complex; it failed to release from the complex to LolA, unless the intermolecular disulfide bond is disrupted by the addition of reducing reagent (Fig 2D). Failure of RcsF release to LolA by the LolC^A106C^DE^S173C^ complex suggests that either an overall conformational change of LolCDE is required or the disulfide bond-fixed PLDs interfere with the lipoprotein transport. In line with our in vitro photo-crosslinking findings, complementation assay also showed that *lolC*^*A106C*^*DE*^*S173C*^ failed to rescue the growth of the *lolCDE*-depleted *E*. *coli* cells unless reducing reagent is supplemented (Figs 2E and S10A). Taken together, we conclude that our apo-LolCDE structure represents the correct apo conformational state of LolCDE, which resembles closely the conformation of RcsF-LolCDE. This study also correlates well with the structural and functional studies of LptB_2_FG [39–42].

### RcsF-LolCDE interactions and lipoprotein substrate selectivity

**Fig 3:**
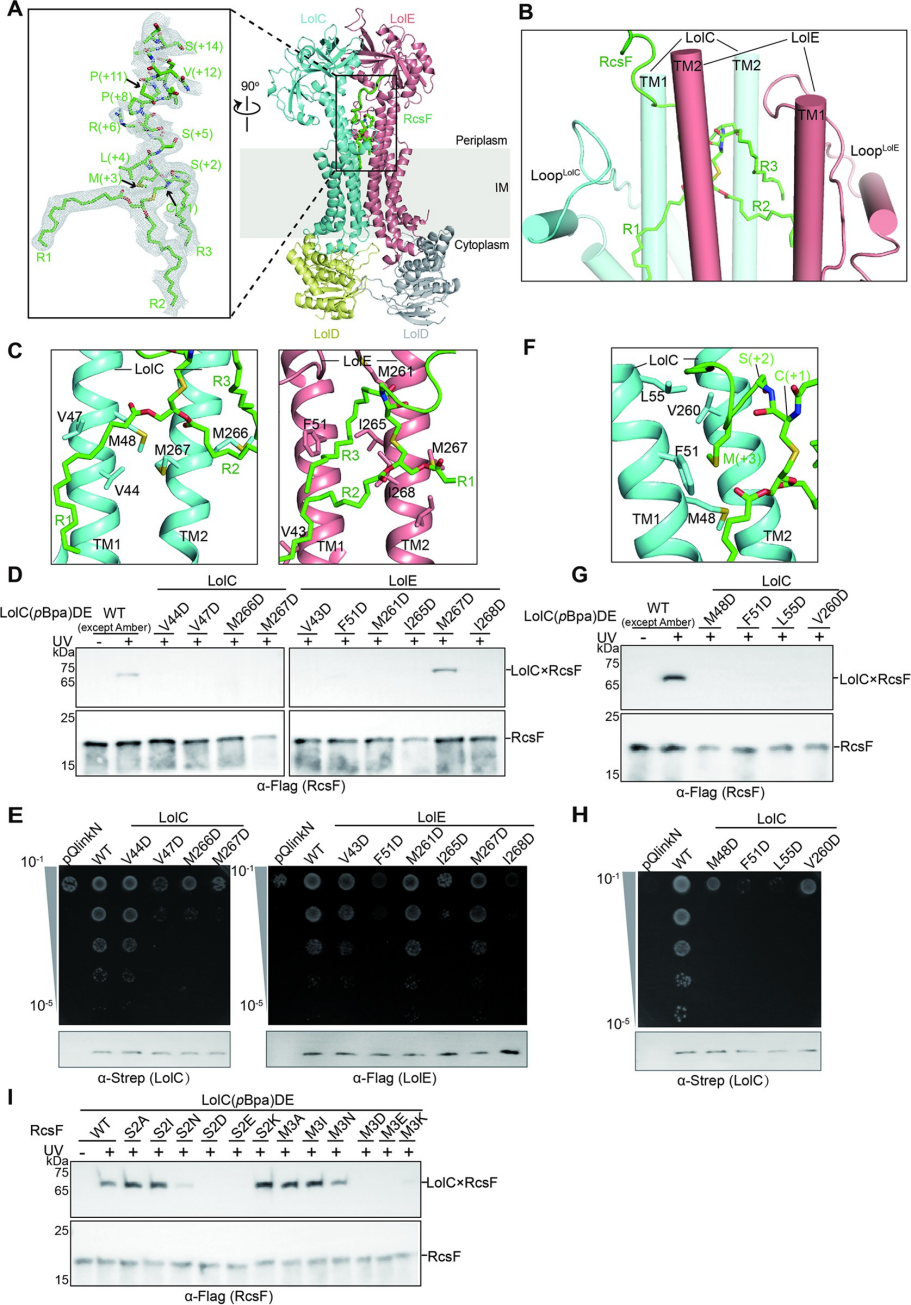
The bipartite binding mode between RcsF and LolCDE. (**A**) Ribbon diagram of RcsF-LolCDE structure (right). Zoom-in view of the atomic model of RcsF superimposed with the cryo-EM densities (left). The arrow indicates the +1 position of the mature RcsF. The 3 acyl chains (R1, R2, and R3) and the N-terminal 14 residues of the proteinaceous portion are labelled. (**B**) Side view of the V-shaped cavity and the RcsF-binding mode. TM segments are shown in cylindrical helices. (**C**) Zoom-in view of the hydrophobic interactions between acyl chains (R1, R2, and R3) and LolC (left) or LolE (right). Residues shown as stick model were substituted with Asp for functional assays. (**D**) Photo-crosslinking assessing the importance of residues of LolCDE that interact with 3 acyl chains of RcsF in (C). *rcsF* were coexpressed with *lolCDE* mutants. (**E**) Complementation assay for the *lolCDE* mutants in (C). Protein leaky expression levels of the *lolC* and *lolE* mutants were detected by western blotting (bottom). (**F**) Zoom-in view of the hydrophobic interactions between Met+3 of RcsF and residues of LolCDE. (**G** and **H**) photo-crosslinking (G) and complementation assays (H) assessing the functional importance of hydrophobic interactions between Met+3 and residues of LolCDE. (**I**) UV-dependent crosslinks between LolCDE and RcsF variants were detected by immunoblotting. Data shown in (D and E) and (G to I) are representatives of 3 replicates.

In our RcsF-LolCDE structure, 3 acyl chains (R1, R2, and R3) and the N-terminal 14 residues of the mature RcsF are well resolved (the first Cys residue of a mature lipoprotein named +1 position) (Fig 3A). Specifically, R1 sits between TM1^LolC^ and TM2^LolE^ (Fig 3B) by making hydrophobic interactions with residues Val44, Val47, and Met48 of TM1^LolC^ and Met267 of TM2^LolE^ (Fig 3C). In the opposite side, R2 and R3, surrounded by TM2^LolC^, TM1^LolE^, and Loop^LolE^ (Fig 3B), are in close contact with residues Met266 and Met267 of TM2^LolC^, Val43, and Phe51 of TM1^LolE^, as well as Met261, Ile265, and Ile268 of TM2^LolE^ (Fig 3C). To probe the functional significance of these contacts, we made point mutations for the abovementioned residues by introduction of hydrophilic residues. We then tested these mutants one by one. Each LolCDE mutant is successfully expressed and assembled giving the right size exclusion chromatography profile (S11A Fig). These mutations (the sole exception being M267D of TM2^LolE^), however, all have their substrate entry severely affected, as inferred from no detectable LolC×RcsF adducts by photo-crosslinking (Fig 3D). Consistent with this, complementation assays also showed that these *lolCDE* mutants supplement failed to fully restore the growth of the *lolCDE*-depleted *E*. *coli* to a varied degree (Figs 3E and S10B). These findings highlight the functional importance of 3 acyl chain-interacting residues in LolCDE and, in turn, corroborate our structural observations.

Of the 14 visible residues of RcsF in the RcsF-LolCDE structure, only 3 residues, Cys+1, Ser+2, and Met+3, are enclosed by the V-shaped cavity of LolCDE (S13A Fig). The remaining residues adopt a stretched loop conformation protruding from the cavity on the Loop^LolC^ side (Fig 3B). First, we tested the functional importance of Met+3-interacting residues in LolC. As shown in Fig 3G, we found that mutations (M48D, F51D, L55D, and V260D of LolC) did not affect the complex assembly (S11B Fig). However, they severely interfered with the entry of RcsF and failed to rescue the growth of the *lolCDE*-depleted *E*. *coli* (Figs 3H and S10C). Next, we tested whether our structural observations agree well with the Lol avoidance signal hypothesis. In line with the hypothesis, we found that S21D (+2 position), S21E (+2 position), as well as a series of hydrophobic-to-hydrophilic mutations at the +3 position of RcsF either abolished or severely affected RcsF entry into the V-shaped cavity of LolCDE (Fig 3I).

Taken together, our structural observations and functional studies reveal a bipartite binding mode of a lipoprotein to LolCDE. The 3 acyl chains and the first 3 residues of a mature lipoprotein dictate its binding affinity to LolCDE and substrate selectivity, respectively, thereby providing a structural explanation for the Lol avoidance signal hypothesis.

### Features of the substrate-binding cavity of LolCDE

**Fig 4:**
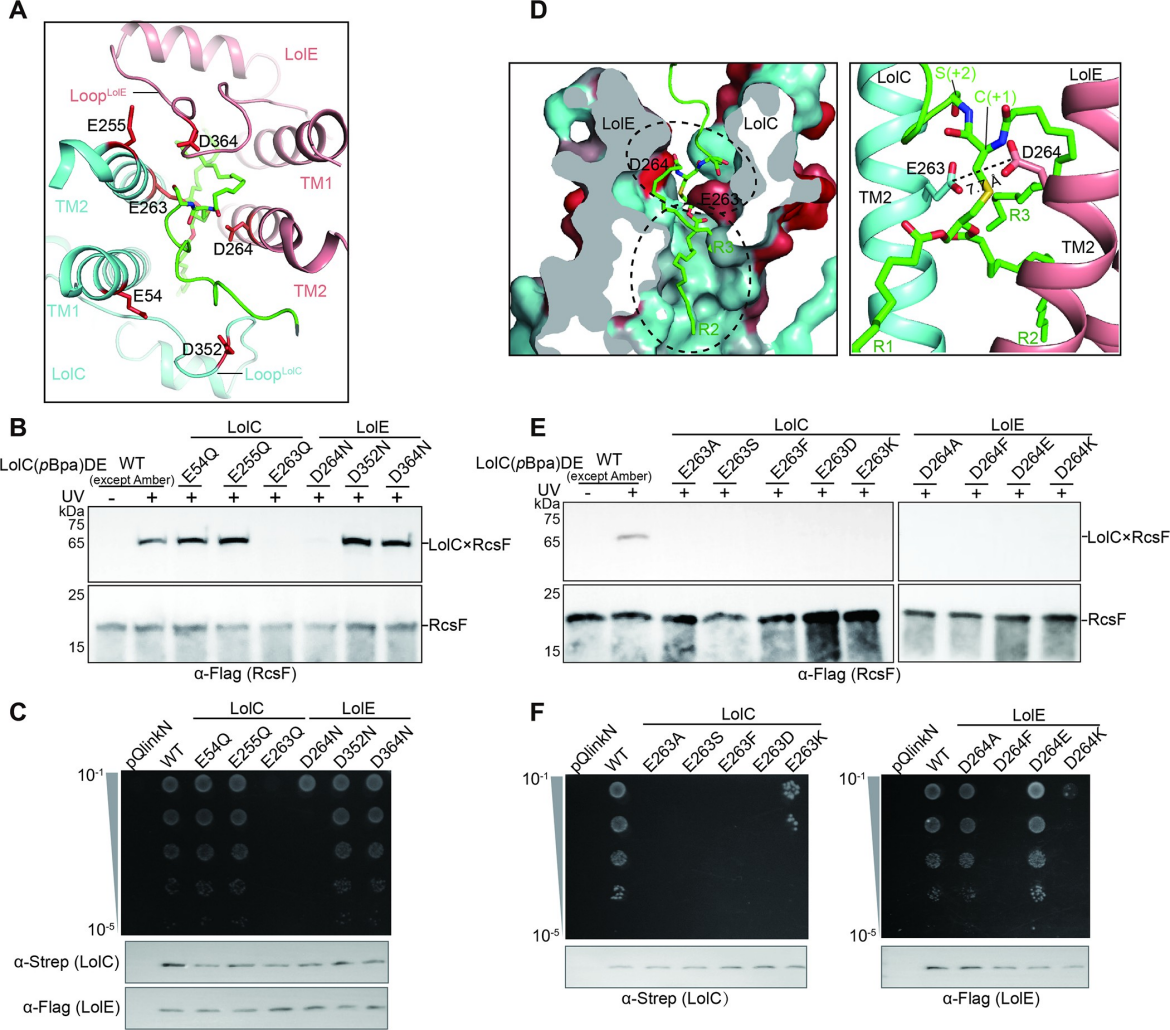
Functional importance of the negatively charged residues in the V-shaped cavity. (**A**) Top view of TMDs of RcsF-LolCDE showing the negatively charged residues (red sticks) lining the upper interior of the V-shaped cavity. (**B** and **C**) The negatively charged residues in (A) were substituted with either Asn or Gln. Photo-crosslinking (B) and complementation assays (C) showing the critical roles of the 2 residues (Glu263^LolC^and Asp264^LolE^) for LolCDE function. (**D**) Cross-sectional view of the hydrophobic surface of the V-shaped cavity, showing the precise positioning of the RcsF into the cavity (left). Hydrophobic and hydrophilic regions of the substrate-binding cavity are colored in blue and red, respectively. Zoom-in view of the location of Cys+1 and Ser+2 of RcsF in the V-shaped cavity (right). The dashed lines and labels indicate the distances between the side chains of Glu263 and Asp264. (**E** and **F**) E263 and D264 were substituted with different types of amino acids for photo-crosslinking (E) and complementation assays (F), respectively. Data shown in (B, C, E, and F) are representatives of 3 replicates.

A prominent feature of the substrate-binding cavity of LolCDE is its negatively charged nature. This property is conferred by 6 acidic residues Glu54, Glu255, Glu263, and Asp352 of LolC together with Asp264 and Asp364 of LolE (Fig 4A). Photo-crosslinking and complementation assays both showed that only 2 of the 6 mutations, E263Q^LolC^ and D264N^LolE^, abolished RcsF entry (Figs 4B and S11) and failed to rescue the growth of the *lolCDE*-depleted *E*. *coli* strain (Figs 4C and S10D). A close examination of the RcsF-LolCDE structure reveals that the side chains of Glu263^LolC^ and Asp264^LolE^ clamp Cys+1 of RcsF from opposite directions and are located right in the center of the cavity (Figs 4D and S13B). Importantly, the distance between the 2 negatively charged groups is only 8.0 Å (Fig 4D). Representative single-point mutations (E263A, E263S, E263F, E263D, and E263K) of LolC abolished RcsF entry (Figs 4E and S11D) and failed to rescue the growth of the *lolCDE*-depleted *E*. *coli* (Figs 4F and S10E). However, for single-point mutations (D264A, D264F, D264E, and D264K) of LolE, only mutations D264F and D264K appeared to be lethal (Figs 4F and S10E), but they all appear to severely interfere with RcsF entry (Figs 4E and S11D). In accordance with these findings, sequence alignment analysis pinpoints Glu263 that is identical in all LolC homologues, with Asp264 highly conserved among LolE homologues (S14 and S15 Figs). The negatively charged nature of the cavity, in particular, the 2 negatively charged residues, Glu263^LolC^ and Asp264^LolE^, which precisely clamp Cys+1 of RcsF, may also play a role in precluding the entry of phospholipids and LPS into the lipoprotein-binding cavity from the IM, for that the negatively charged PO_4_^−^ groups of phospholipids and LPS in the membrane are similarly positioned as Cys+1 of a lipoprotein [40,44–48] (S13C Fig).

**Fig 5:**
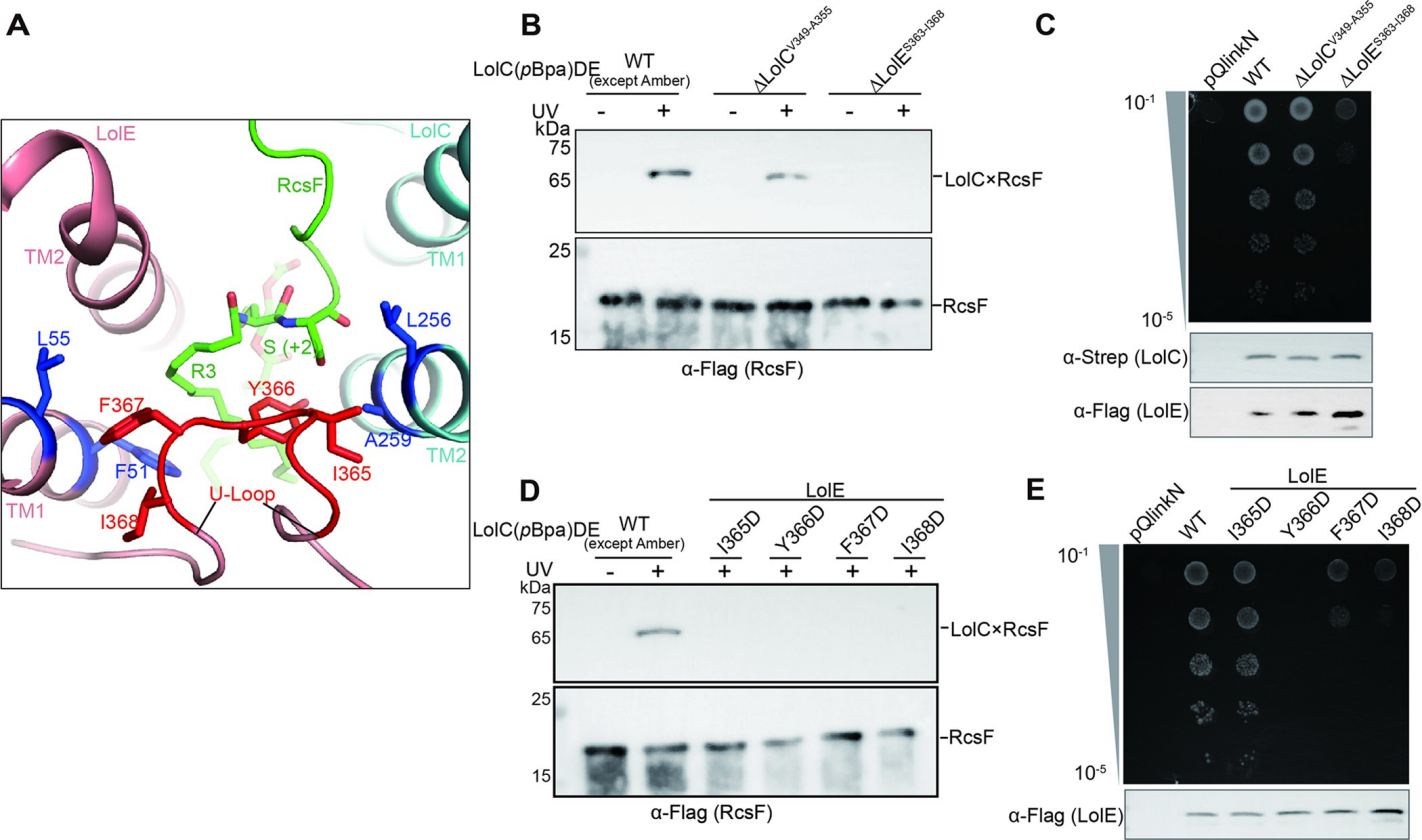
The U-loop maintains the configuration of the substrate-binding cavity. (**A**) Top view of the V-shaped cavity showing that the U-Loop (red) interacts with TMs. Residues from both TM1^LolE^ and TM2^LolC^ (in blue) make hydrophobic contacts with the U-Loop (red). (**B** and **C**) The 6 residues (S363-I368 of LolE) that consist of the U-Loop and the 7 residues (V349-A355 in Loop^LolC^) were deleted, respectively. Photo-crosslinking (B) and complementation assays (C) showing that the U-Loop is crucial for LolCDE function. (**D** and **E**) Residues (in red stick model) in (A) were substituted with Asp for photo-crosslinking (D) and complementation assays (E) respectively. Data shown in (B to E) are representatives of 3 replicates.

A second distinct feature of the substrate-binding cavity is that the U-Loop from the Loop^LolE^ plugs into the TM1^LolE^-TM2^LolC^ interface, making hydrophobic contacts with residues from both TM1^LolE^ and TM2^LolC^ (Fig 5A). By contrast, in the AMPPNP-LolCDE structure, the substrate-binding cavity is compressed with the U-Loop completely squeezed out (S8C Fig). As the sequences of both LolC homologues and LolE homologues are highly conserved (S14 and S15 Figs), and both LolC and LolE adopt a rather similar fold (S7B and S7C Fig), we speculated that the U-Loop, which is absent in Loop^LolC^, may participate in lipoprotein entry. To test this hypothesis, we deleted 6 residues of the U-Loop (residues Ser363-Ile368 of LolE) and tested the entry of RcsF into the V-shaped cavity. As shown in Fig 5, the deletion mutant abolished RcsF entry. By contrast, a 7-residue deletion (residues Val349-Ala355 of LolC) in Loop^LolC^ showed no noticeable effects on RcsF entry (Figs 5B and 5C and S10F and S11E). Furthermore, any hydrophobic-to-hydrophilic mutations (I365D, Y366D, F367D, and I368D) in the U-Loop also caused LolCDE loss-of-function to varied degrees (Figs 5D and 5E and S10 and S11). These results argue for the U-Loop to play a role in maintaining the configuration of the V-shaped cavity.

### A single path for lipoprotein entry into the substrate-binding cavity

**Fig 6:**
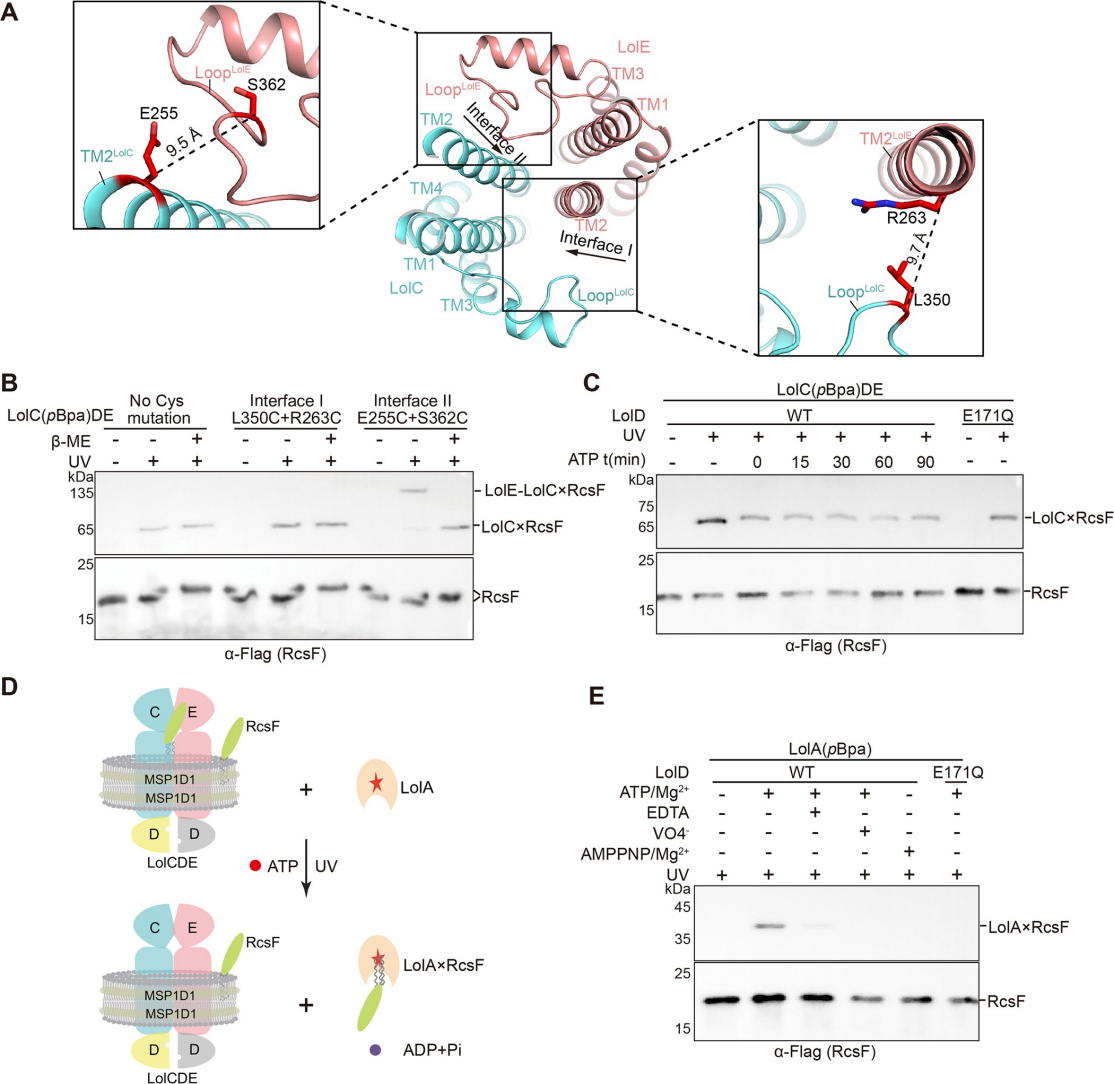
A single path for lipoprotein entry into the V-shaped cavity and energy requirement for lipoprotein transfer to LolA. (**A**) Top view of 2 potential gates (Interface I and Interface II) for lipoprotein entry (middle). Zoom-in view of 2 pairs of residues replaced with cysteines in (B). (**B**) In vitro photo-crosslinking. LolC^L256*p*BPA^DE that contain 2 cysteine mutations or not were reconstituted with RcsF in nanodisc. The LolE-LolC×RcsF adducts were detected by immunoblotting. (**C**) LolC^L256*p*BPA^DE that contain either wild-type LolD or LolD (E171Q) were reconstituted with RcsF in nanodisc. The adducts were evaluated by exposing to UV radiation with or without addition of ATP and Mg^2+^. (**D**) Scheme of an in vitro one-cycle lipoprotein transfer to LolA. Addition of LolA (W70*p*BPA), along with ATP and Mg^2+^, leads to transfer of RcsF from LolCDE to LolA. (**E**) Nanodisc-embedded RcsF-LolCDE proteins that contain either wild-type LolD or LolD (E171Q) were incubated with LolA (W70*p*BPA) and nucleotides. The ability to transfer RcsF to LolA (W70*p*BPA) from LolCDE was probed. Data shown in (B, C and E) are representatives of 3 replicates.

As mentioned above, the substrate-binding cavity of apo-LolCDE has 2 intermolecular interfaces, the Loop^LolC^-TM2^LolE^ interface (denoted as Interface I) and the Loop^LolE^-TM2^LolC^ interface (denoted as Interface II) (Fig 6A), which can both serve as potential gates for lipoprotein entry. To figure out the exact entry route, we introduced intermolecular disulfide bonds to block 1 gate and probed the entry of RcsF via the other. In each case, however, we obtained only partial formation of disulfide bonds in the LolCDE samples; we therefore proceed focusing on the crosslinking of RscF and LolC-LolE, those crosslinks that do contain intermolecular disulfide bonds between LolC and LolE. A potential disulfide bond between E255C^LolC^ and S362C^LolE^ in LolCDE was first introduced in hope to prevent RcsF entry via the Interface II, and SDS-PAGE analysis revealed that, though not complete, disulfide bonds were formed in the LolC^L256*p*BPA+E255C^DE^S362C^ complexes (S13D Fig). After reconstitution into nanodisc together with Flag-tagged RcsF, the LolE-LolC×RcsF adducts were clearly detected under nonreducing condition upon UV radiation (Figs 6B and S16 and S1 Data). This demonstrated that formation of intermolecular disulfide bonds in the LolCDE complexes blocks the Interface II; however, the RcsF entry into the V-shaped cavity is unaffected. This strongly suggests that Interface I serves as the substrate entry route. Similarly, disulfide bond between L350C^LolC^ and R263C^LolE^ was introduced in hope to block Interface I. Again, intermolecular disulfide bonds formation was incomplete in the LolC ^L256pBPA+L350C^DE^R263C^ complexes (S13D Fig), and no LolE-LolC×RcsF crosslinks were detected after in vitro photo-crosslinking (Fig 6B). Absence of LolE-LolC×RcsF crosslinks strongly implicates that RcsF is unable to enter the cavity with Interface I blocked. Base on the results, we propose that lipoproteins enter the cavity via Interface I rather than Interface II of LolCDE. These findings correlate well with our previous claim that the U-Loop in the Loop^LolE^ takes part in buttressing the substrate-binding cavity in an outward conformation, and with the structural observation that the proteinaceous portion of RcsF is only located on the Loop^LolC^ side in the RcsF-LolCDE structure. Results from our experiments are self-consistent and in line with structural observations.

### Lipoprotein entry is ATP-independent, but its transport to LolA requires ATP hydrolysis

To investigate which step of the lipoprotein transport cycle requires ATP, we performed in vitro lipoprotein transfer assays under different conditions. First, we probed how ATP affects the entry of RcsF into the V-shaped cavity by photo-crosslinking. Apparently, addition of ATP, regardless of incubation time, did not increase the amounts of the LolC×RcsF adducts (Fig 6C). The relatively reduced LolC×RcsF adducts upon addition of ATP may result from the formation of the ATP-bound LolCDE complexes that had precluded the entry of RcsF as indicated by the AMPPNP-LolCDE structure. Importantly, a catalytically dead mutant LolCD^E171Q^E allowed the entry of RcsF (Fig 6C), strongly supporting the notion that lipoprotein entry is ATP-independent. Next, we incorporated *p*BPA at Trp70 that is located at the interior of LolA (S13E Fig) and performed lipoprotein transfer assays (Fig 6D). Clearly, LolA (W70*p*BPA) was able to crosslink to RcsF only in the presence of ATP and Mg^2+^ (Fig 6E). Conditions that interfere with ATP hydrolysis, e.g., addition of EDTA, VO_4_^−^ or nonhydrolysable AMPPNP, as well as the catalytically dead mutant LolCD^E171Q^E protein, all failed to produce LolA×RcsF adducts (Fig 6E). However, it appears that the ATPase activity of LolCDE decreases with the addition of lipoproteins, which can be stimulated by LolA (S8E Fig and S1 Data). Taken together, our studies demonstrate that lipoprotein transfer to LolA from LolCDE requires ATP hydrolysis, and efficient substrate release increases the ATPase activity of LolCDE.

## Discussion

**Fig 7:**
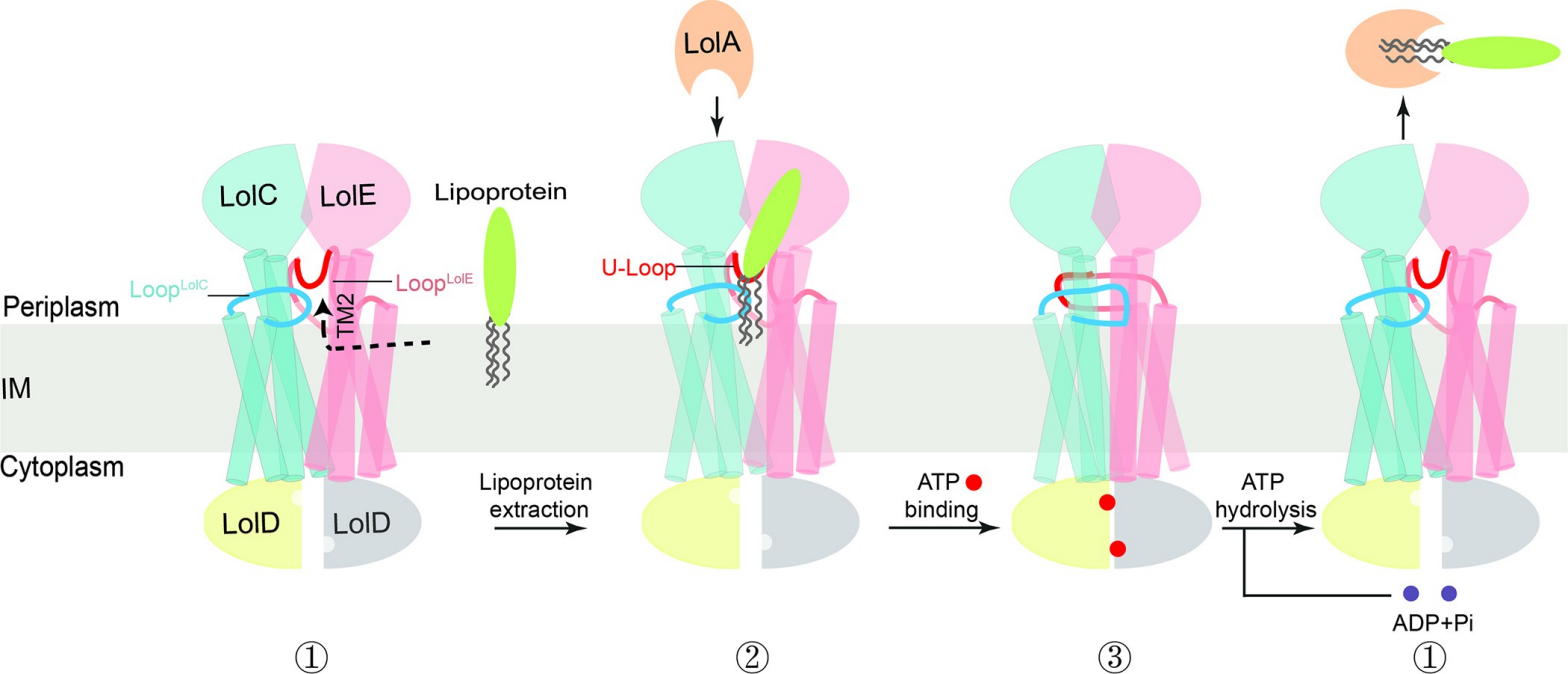
Proposed model for lipoprotein extraction and transfer to LolA. ATP and ADP are shown as balls in red and purple, respectively. The dashed arrow indicates the path for lipoproteins entry into the cavity. Our apo-LolCDE, RcsF-LolCDE and AMPPNP-LolCDE structures represent structures in ①, ②, and ③, respectively.

On the basis of the structural and functional results reported here, we proposed a working model for lipoprotein transport by LolCDE (Fig 7). First, apo-LolCDE embedded in the inner membrane adopts an outward-facing conformation. The open conformation of apo-LolCDE allows lipoprotein entry into the substrate cavity via the Loop^LolC^-TM2^LolE^ interface (i.e., the Interface I) in an ATP-independent manner. The bound lipoprotein has its first 3 residues encompassed by the substrate-binding cavity, the remainder bulging into the periplasm on the Loop^LolC^ side. Second, ATP binding causes a tight dimerization of LolD and closure of V-shaped cavity, pushing the bound lipoprotein out of the cavity. The final step involves ATP hydrolysis followed by conformational changes that enable lipoprotein transfer from LolCDE to LolA. Upon release of the LolA-lipoprotein complex and ADP, LolCDE returns to its apo-state primed for the next transport cycle.

While LolCDE, a type VII ABC transporter, differs from the type VI ABC transporter LptB_2_FG in both substrates and transmembrane topologies [39,40,42], they do share similarities in a number of aspects. First, lipoproteins enter and lodge into the substrate-binding cavity requiring no input of energy. Rather, substrate delivery to LolA downstream in the transport cycle is driven by the energy from ATP hydrolysis. Second, substrate binding does not cause any conformational changes of the ABC transporter. Third, substrate entry into the binding cavity proceeds via one particular side of the TMD interface of the ABC transporter. The combined structural evidences bear strong implications that underlines a mechanotransmission mechanism utilized by LolCDE for substrate extrusion and delivery, similar to the type VII ABC transporter MacB [39,40,42] (S17 Fig). In this regard, it is interesting to note that the remarkable resemblance of the overall architectures of apo- and nucleotide-bound LolCDE and MacB, the only exception being that apo-MacB lacks the V-shaped substrate-binding cavity and a central channel through which substrates take path [37,38].

Collectively, here, we report 3 LolCDE structures, apo, RcsF-, and AMPPNP-bound states. While the latter two correlate well with the previously published structures reported by Tang and colleagues and Sharma and colleagues, the former deviates significantly from the apo-LolCDE* structure reported by Tang and colleagues [33,34]. The functional analysis confirms our apo-LolCDE structure, highlighting that lipoproteins enter and bind in the cavity of apo-LolCDE devoid of apparent conformational changes in an ATP-independent manner. Furthermore, our high-quality structure of RcsF-LolCDE reveals a bipartite binding mode of a lipoprotein with LolCDE, as well as a functional role of the U-loop in maintaining the configuration of the V-shaped cavity. In particular, we identified a single path of lipoprotein-entry into the LolCDE. Taken together, our results provide deep insights into the mechanisms underlying LolCDE-mediated lipoprotein sorting in *E*. *coli*. Since lipoprotein sorting is essential for the survival of gram-negative bacteria [10], the RcsF-LolCDE structure we presented here also promises to guide novel antibiotic therapies fighting against drug resistant pathogens.

## Materials and methods

### Cloning, expression, and purification of the LolCDE, RcsF-LolCDE, and LolCD^E171Q^E complexes

The 3 gene fragments containing *lolC* with NcoI/XbaI, *lolD* with XbaI/KpnI, and *lolE* with KpnI/HindIII were amplified individually from *E*. *coli* K-12 MG1655 genomic DNA by PCR. The 3 fragments were subsequently ligated into the pBAD22 vector. The recombinant plasmid pBAD22**-**LolCDE, including a C-terminal Strep-tag II on LolD, was used to transform *E*. *coli* BL21 (DE3) for LolCDE expression. The bacterial cells were grown at 37°C in Luria broth (LB) medium with 100 μg/ml ampicillin sodium until the optical density of the culture reached 1.0 at 600 nm. Protein expression was induced with 0.05% w/v L-arabinose at 18°C. After 14-h expression, cells were collected by centrifugation at 4,000 rpm for 20 min at 4°C and resuspended in buffer containing 20 mM Tris-HCl (pH 8.0), 150 mM NaCl. Resuspended cells were lysed by sonication (MisonixSonicator S-4000; Cole Parmer, Vernon Hills, IL, USA), and then centrifuged at 18,000 rpm (rotor ID: JA25.50, Avanti Centrifuge, J-26XP, Beckman Coulter) for 1 h at 4°C to collect the total cell membranes. The total membranes were solubilized in 20 mM Tris-HCl (pH 8.0), 150 mM NaCl, 10% v/v glycerol, and 1% w/v n-dodecyl-β-d-maltoside (DDM; Anatrace) at 4°C for 1 h. The supernatants were collected after centrifugation at 18,000 rpm for 1 h at 4°C and incubated with preequilibrated Strep-Tactin beads (DiNing) for 30 min at 4°C. After rinsing the Strep-Tactin beads with wash buffer (20 mM Tris-HCl (pH 8.0), 150 mM NaCl, 3% glycerol, and 0.05% DDM), the LolCDE protein was eluted using wash buffer containing 2.5 mM d-Desthiobiotin (Sigma).

To obtain the RcsF-LolCDE complex, the gene fragment containing *rcsF* was amplified from *E*. *coli* K-12 MG1655 genomic DNA and ligated into pET28a vector. Plasmids pBAD22**-**LolCDE and pET28a-RcsF were then cotransformed into *E*. *coli* BL21 (DE3) for overexpression. Expression and purification of the RcsF-LolCDE and the catalytically dead LolCD^E171Q^E complexes followed a similar protocol for the LolCDE complex as described above.

### Nanodisc reconstitution

POPG (Avanti Polar Lipids) was solubilized in chloroform and dried under nitrogen to form a thin lipid film. The lipid film was hydrated and resuspended at a concentration of 25 mM POPG in 250 mM sodium cholate. LolCDE, LolCD^E171Q^E, or RcsF-LolCDE complex, MSP1D1 membrane scaffold protein [49], and POPG were mixed at a molar ratio of 1:2.4:80 in a buffer containing 15 mM sodium cholate and incubated for 30 min at 4°C. Detergents were removed by incubation with 0.8 mg/ml Bio-Beads SM2 (Bio-Rad) overnight at 4°C. Nanodisc-embedded LolCDE, LolCD^E171Q^E, and RcsF-LolCDE complexes were further purified using a Superose6 increase 10/300GL column (GE Healthcare) in a buffer containing 20 mM Tris-HCl (pH 8.0) and 150 mM NaCl.

### Cryo-EM specimen preparation, data acquisition, and processing

To prepare samples for cryo-EM analysis, 3 μl of nanodisc-embedded LolCDE, LolCD^E171Q^E, or RcsF-LolCDE complex at a concentration of 0.6 mg/ml was applied to glow-discharged Quantifoil holey carbon grids (2/2, 300 mesh). For AMPPNP/Mg^2+^ trapping, the samples were incubated with 2 mM AMPPNP (Sigma) and 2 mM MgCl_2_ for 30 min at 4°C before applying the samples to cryo-EM grids. Grids were blotted for 5 s with 75% relative humidity and plunge-frozen in liquid ethane cooled by liquid nitrogen using automatic plunging device EMGP (Leica). Cryo-EM data set of LolCDE and RcsF-LolCDE complexes were collected in a 300-KV Titan Krios G2 microscope (Thermal Fisher) equipped with a direct detector K2 camera (Gatan) and GIF quantum energy filter (energy width of 20 eV). The data set of AMPPNP-bound LolCD^E171Q^E was collected in a 200-KV Talos Arctica microscope (Thermal Fisher) equipped with direct detector K2 camera (Gatan) and GIF quantum energy filter (energy width of 20 eV) using beam-image shift data collection method [50]. Micrographs of LolCDE, RcsF-LolCDE and LolCD^E171Q^E were recorded at a defocus range of −1.8 to −2.2 μm with a pixel size of 1.36 Å, 1.04 Å, and 1.0 Å, respectively. The beam-induced motion of each micrograph stack was corrected by MotionCor2 [51], and the defocus parameter of each micrograph was determined by CTFFIND4 [52]. For LolCDE data set, 134,138 particles were picked from 300 micrographs using the Laplacian-of-Gaussian method in Relion-3.0. Five good classes were selected after 2D classification and were used as the references for automatic particle picking from all micrographs. A total of 2,666,570 particles were picked and subjected to 2D classification. After 3 rounds of 2D classification, 976,646 particles were kept for further data processing. The initial model for 3D classification was generated from selected particles using the SGD initial model generation program in Relion-3.0. After 3D classification, 503,068 particles were selected and subjected to 3D refinement, CTF refinement, and particle polishing, yielded a 4.0-Å EM map in Relion-3.0. For RcsF-LolCDE and AMPPNP-LolCD^E171Q^E data sets, a similar process procedure was applied in Relion-3.0 [53]. Then, 225,933 and 277,296 good particles were transferred to CryoSPARC-2.14 [54]. The 3.5-Å and 3.6-Å maps for RcsF-LolCDE and AMPPNP-LolCD^E171Q^E were, respectively, generated after NU-refinement, particle subtraction, and local refinement with a soft mask on protein parts.

To obtain the apo-LolCDE map from LolCDE dataset, 503,068 particles that yielded 4.0-Å map were subjected to further multireference 3D classification with references generated from this map by multiply soft masks that includes lipoprotein densities or not. The references were initial low-pass filtered to 15 Å and an angular sampling of 3.7° was combined with local angular searches in the classifications. After several rounds of 3D multireference classification, 135,391 particles yielded a 4.2-Å apo-LolCDE map with no lipoprotein densities in the cavity. The other 367,675 particles yielded a 4.1-Å lipoprotein-LolCDE map with strong densities of lipoproteins in the cavity. To avoid reference bias, the reclassified maps were all reconstructed with the alignment file of the previous 4.0-Å map. For validation, we generated 2 references that one included no densities of lipoprotein and the other included the densities of proteinaceous parts but not the 3 acyl chains of lipoprotein, then reconstructed reclassified particles after classification with the 2 references to generate 2 maps. The densities of proteinaceous parts and the 3 acyl chains of lipoprotein are all still in 1 map and not in the other one (S2G Fig). In the same way, we also did classifications with 2 references that one of them lacked the densities of Loop^LolE^ or partial TM2^LolE^. The maps generated from the 2 different classifications both have the densities lacked in references (S2H and S2I Fig). Taken together, this supported that there was no reference bias in our reclassification method and the map of apo-LolCDE was correct.

### Model building and refinement

To build the model of the RcsF-LolCDE complex, the crystal structures of the PLD of LolC from *E*. *coli* (PDB 5NAA), the PLD of LolE from *Acinetobacter baumannii* (PDB ID: 5UDF), and LolD from *Aquifexaeolicus* (PDB ID: 2PCL) were fitted into the cryo-EM map in UCSF Chimera [55]. After changing all amino acids to the correct *E*. *coli* sequence and manually building the TMDs of LolC and LolE in Coot [56], the initial model was refined using real space refinement in PHENIX and manually adjusted in coot. For building the model of AMPPNP-LolCD^E171Q^E, the previous structure of RcsF-LolCDE was used as the starting model and fitted into the 3.6-Å map by model morphing in coot [57]. Two AMPPNP molecules and Mg^2+^ are manually fitted into the 2 additional densities at the LolD molecules. The model was refined using real space refinement in PHENIX, subsequently. The atomic model of the 4.2-Å apo LolCDE was built using the structure of RcsF-LolCDE as a starting model, and the RcsF atoms that lacked relevant density were manually deleted. The structural model was refined using real space refinement in PHENIX [58]. Cryo-EM data collection and refinement statistics are summarized in S1 Table.

### ATPase activity assay

The ATPase activity assays were performed using Malachite Green Phosphate Assay Kits (Bioassay Systems). The phosphate standards and blank control for colorimetric detection were prepared according to the manufacturer’s instructions. Approximately 0.2 μM LolCDE, RcsF-LolCDE, or LolCDE mutant protein in nanodisc were incubated with 2 mM ATP and 2 mM MgCl_2_ at room temperature for 15 min directly or incubated with LolA at room temperature for 10 min before incubated with ATP. Since the ATP concentration should be lower than 0.25 mM, the mixture was diluted 1:10 in buffer containing 20 mM Tris-HCl (pH 8.0), 150 mM NaCl. Each 80 μL sample solution was transferred into a separate well of the plate, and 20 μL of the working reagent was added. The mixtures were incubated for 30 min at room temperature for color development. The absorbance at 620 nm was measured. The ATPase activities of all samples were determined using the mean value of the samples according to linear regression for the standards. All experiments were repeated at least 3 times.

### Detection of RcsF-LolCDE interactions by photo-crosslinking

*E*. *coli* BL21 (DE3) strains were cotransformed with pSup-BpaRS-6TRN22 and pBAD22**-**LolCDE (or *lolCDE* mutant), and pET28a-RcsF with a C-terminal Flag tag (or *rcsF* mutant). An amber (TAG) codon was introduced at L256^LolC^ in pBAD22-LolCDE. Cells were grown at 37°C in LB containing 30 μg/ml ampicillin sodium, 15 μg/ml kanamycin, and 15 μg/ml chloramphenicol in dark. When the cultures reached OD 600 ~ 1.0, 0.5 mM *p*BPA was added into LB. After 1 h, protein expression was induced by the addition of 0.1 mM IPTG and 0.05% L-arabinose at 18°C for 14 h. Purification of RcsF-LolC^L256*p*BPA^DE complex was performed using a similar protocol for LolCDE. Protein samples were concentrated to 0.6 mg/ml using a 100-kDa cutoff spin concentrator (Millipore), then either used for western blot analysis directly or exposed to UV light (365 nm, 100 W; Thermo Fisher Scientific) for 10 min before western blot analysis.

For western blot analysis, the samples were mixed with SDS loading buffer (containing 5 mM β-ME). The protein samples were separated by SDS-PAGE (12%) and subsequently transferred to PVDF membranes (Bio-Rad). The PVDF membrane containing proteins was blocked using TBST buffer (20 mM Tris-HCl (pH 8.0), 150 mM NaCl, 0.1% Tween-20) containing 8% skim milk for 1 h. The PVDF membrane was then incubated with Flag tag antibody (1:3,000 dilution, AP1013a, YTHX, China) at room temperature for 1 h and subsequently washed with TBST buffer 3 times and further incubated with goat anti-rabbit horseradish peroxidase (HRP)-conjugated secondary antibody (1:5,000 dilution, LABLEAD, China) at room temperature for 1 h. LolC/E-RcsF adducts were visualized using an enhanced chemiluminescence detection kit (Applygen, China).

### In vitro photo-crosslinking assay

pET28a-RcsF with a C-terminal Flag-tag was transformed into *E*. *coli* BL21 (DE3) strain for RcsF expression. The bacterial cells were grown at 37°C in LB with 35 μg/ml kanamycin until the optical density of the culture reached 1.0 at 600 nm. Protein expression was induced with 0.1 mM IPTG at 25°C for 12 h. Purification of RcsF was performed using a similar protocol for LolCDE, with slight modifications. The supernatants were incubated with anti-Flag affinity gel (YESEN) and eluted with wash buffer containing 100 μg/ml 3×Flag-tag peptide (YESEN). The protein samples were concentrated to 0.4 mg/ml using a 30-kDa cutoff spin concentrator. LolC^L256*p*BPA^DE or cysteine mutations were purified using a similar protocol for LolCDE. RcsF, LolC^L256*p*BPA^DE or cysteine mutations, MSP1D1 membrane scaffold protein and POPG were mixed at a molar ratio of 1:1:2.4:80. The nanodisc-embedded complexes were applied to a Superose6 increase 10/300GL column preequilibrated in buffer containing 20 mM Tris-HCl (pH 8.0) and 150 mM NaCl. Peak fractions were combined and concentrated to 0.6 mg/ml, then either used for western blotting directly or exposed to UV light for 10 min before western blotting. For western blotting analysis, samples were mixed with SDS loading buffer and analyzed by SDS-PAGE and western blotting with anti-Flag antibody as described above, but β-ME was not added to samples.

### In vitro lipoprotein transfer assay

To purify LolA(W70*p*BPA) for lipoprotein transfer assay, plasmids pBAD22-LolA (W70*p*BPA) with a C-terminal His-tag and pSup-BpaRS-6TRN22 were cotransformed into *E*. *coli* BL21 (DE3) strain. The bacterial cells were grown at 37°C in LB with 100 μg/ml ampicillin sodium and 15 μg/ml chloramphenicol in dark. When the cultures reached OD600 ~ 0.8, 0.5 mM *p*BPA was added into LB. After 1 h, protein expression was induced with 0.05% L-arabinose at 25°C for 12 h. After centrifugation, cells were resuspended in buffer containing 20 mM Tris-HCl (pH 8.0), 150 mM NaCl, 20 mM imidazole and lysed by sonication. The supernatants were collected after centrifugation at 18,000 rpm for 1 h at 4°C and incubated with preequilibrated Ni-NTA agarose beads for 1 h at 4°C. After rinsing the Ni-NTA agarose beads with wash buffer containing 20 mM Tris-HCl (pH 8.0), 150 mM NaCl, and 50 mM imidazole, the LolA (W70*p*BPA) protein was eluted using wash buffer containing 300 mM imidazole. The protein samples were concentrated to 0.5 mg/ml using a 30-kDa cutoff spin concentrator.

To perform lipoprotein transfer assay, the nanodisc-embedded RcsF-LolCDE (or RcsF-LolCD^E171Q^E) and LolA (W70*p*BPA) were mixed at a molar ratio of 1:1. The mixtures were incubated with 5 mM ATP/Mg^2+^, 5 mM ATP/Mg^2+^/EDTA, 5 mM ATP/Mg^2+^/VO4−, or 5 mM AMPPNP/Mg^2+^ at room temperature for 15 min or set aside and exposed to UV light for 10 min before western blotting.

### Construction of the *lolCDE*-depleted *E*. *coli* strain

The *lolCDE*-depleted strain was constructed based on *E*. *coli* strain BW25113 with deletion of *lpp* gene and integration of a terminator and an arabinose-inducible *araBAD* into the chromosome prior to the *lolCDE* locus, using CRISPR-Cas9 system [59]. In brief, a 20-nt single guide RNA (sgRNA) targeting *lpp* gene was introduced in pTargetF plasmid. The BW25113 strain carrying pCas plasmid was cotransformed with the pTargetF-*lpp* and a donor DNA fragment that including 300 nt from each side of *lpp* gene. The *lpp*-deleted strain BW25113-DL was selected by PCR and confirmed by sequencing. As the same strategy, a 20-nt sgRNA targeting the *lolCDE* gene was introduced in pTargetF plasmid. An rrnB_T1-rrnB_T2 terminator was inserted in front of P*araBAD*, and the donor DNA fragment carrying rrnB_T1-rrnB_T2-P*araBAD* and 500 nt homologous nucleotides was cotransformed into BW25113-DL carrying pCas and pTarget-*lolCDE*. The *lolCDE*-depleted strain BW25113-DLDL was selected by PCR and confirmed by sequencing.

### Complementation assay

The *lpp*-deleted and *lolCDE*-depleted BW25113-DLDL cells harboring either the pQLink-*lolCDE* plasmid [60] or a plasmid encoding a LolCDE mutant protein were plated on LB-agar plates with 100 ug/ml ampicillin and 0.2% L-arabinose. Single colony was picked up and inoculated into 5 mL LB with 100 ug/ml ampicillin and 0.05% L-arabinose. When cells grew to a density of OD600 ~ 1.0, they were collected by centrifugation and washed with 5 mL LB medium twice to remove L-arabinose. The washed cells were resuspended in different volumes of LB medium to ensure that each sample has the same starting cell density of OD600 ~ 0.5 and was subsequently serially diluted (1:10, 1:100, 1:1,000, 1:10,000, and 1:100,000), and 2 μl of each dilution was spotted on LB plates containing 100 ug/ml ampicillin with or without 0.2% L-arabinose. The BW25113-DLDL cells transformed with the empty plasmid pQLinkN were used as a negative control, while the cells harboring the wild-type pQLink-*lolCDE* plasmid was used as a positive control.

Western blot was performed to compare the protein expression levels of the wild-type *lolCDE* and its mutants. LolC and LolE were tagged with Strep and Flag, respectively. Anti-Strep II tag antibody (1:3,000) and anti-Flag antibody (1:3,000) were used to detect LolC and LolE with HRP-conjugated anti rabbit antibody (1:5,000), respectively. Complementation assays were performed in triplicate and a representative result is shown.

## Supporting information

S1 Fig Purification and reconstitution of LolCDE and RcsF-LolCDE in nanodisc. (**A** to **C**), Representative size-exclusion chromatography profiles and Coomassie blue–stained SDS–PAGE gel analysis of LolCDE **(A)**, RcsF-LolCDE **(B),** and LolCD^E171Q^E **(C)** in nanodisc. (**D**) Silver staining of LolCDE and RcsF-LolCDE samples that were used for structure determination. RcsF was copurified LolCDE; LolD* is the degraded LolD band. (**E**) Mass spectrometry identification of RcsF peptide (IYTNAEELVGKPFR). The experiments in (A to C) were repeated over 20 times.
(PNG)

S2 Fig Cryo-EM data processing and analysis of apo-LolCDE. (**A**) Representative cryo-EM micrograph of LolCDE in nanodisc. (**B**) Selected 2D class averages of cryo-EM particle images. (**C**) Scheme of 3D classification and refinement of cryo-EM particle images. (**D**) Gold-standard Fourier shell correlation (FSC) curves of the final cryo-EM maps of LolCDE (4.2 Å) and lipoprotein-LolCDE (4.1 Å). The resolutions were determined at FSC = 0.143. (**E**) The cryo-EM maps of apo-LolCDE (left) and lipoprotein-LolCDE (right). The lipoprotein densities are colored in green. (**F**) Top-down view of the slice through the cryo-EM map of apo-LolCDE (left) and lipoprotein-LolCDE (right), as indicated by the black dotted line in (**E)**. The extra densities of lipoprotein (green) were only observed in the slice through the cryo-EM map of lipoprotein-LolCDE. (**G**) Verification classification with 2 references that one excluded RcsF densities and the other excluded the densities of 3 acyl chains but not the RcsF proteinaceous parts, showing that extra densities of the proteinaceous parts and 3 acyl chains both existed in 1 map. (**H**) Verification classification with 2 references that one excluded the densities of the Loop^LolE^, showing that the densities of Loop^LolE^ lacked in reference still existed in the final maps. (**I**) The verification classification with 2 references that one excluded the densities of partial TM2^LolE^, showing the densities of partial TM2^LolE^ lacked in reference still existed in the final maps.
(PNG)

S3 Fig Cryo-EM data processing and analysis of the RcsF-LolCDE. **(A**) Representative cryo-EM micrograph of RcsF-LolCDE in nanodisc. (**B**) Selected 2D class averages of cryo-EM particle images. (**C**) Scheme of 3D classification and refinement of cryo-EM particle images. (**D**) Gold-standard FSC curves calculated with different masks in cryoSPARC. The resolutions were determined at FSC = 0.143 (horizontal blue line). The final corrected mask gave an overall resolution of 3.5 Å. **(E**) Distribution of orientations over azimuth and elevation angles for particles included in the calculation of the final map. (**F**) Cryo-EM map of RcsF-LolCDE colored by local resolution.
(PNG)

S4 Fig Cryo-EM densities superimposed with the atomic model for selected regions in RcsF-LolCDE. (**A** to **D**) Cryo-EM densities superimposed with the atomic model for TMs of LolC (**A**), TMs of LolE (**B**), RcsF(**C**), and Loop^LolE^ (**D**), respectively.
(PNG)

S5 Fig Cryo-EM data processing and analysis of AMPPNP-LolCDE. (**A**) Representative cryo-EM micrograph of AMPPNP-LolCDE in nanodisc. (**B**) Selected 2D class averages of cryo-EM particle images. (**C**) Scheme of 3D classification and refinement of cryo-EM particle images. (**D**) Gold-standard FSC curves calculated with different masks in cryoSPARC. The resolutions were determined at FSC = 0.143 (horizontal blue line). The final corrected mask gave an overall resolution of 3.6 Å. (**E**) Distribution of orientations over azimuth and elevation angles for particles included in the calculation of the final map. (**F**) Cryo-EM map of AMPPNP-LolCDE colored by local resolution.
(PNG)

S6 Fig Cryo-EM maps of LolCDE in different states. (**A**) Location of the 2 AMPPNP (red spheres) and Mg^2+^ (green spheres) molecules clamped between the 2 LolD subunits (left). Cryo-EM densities (deep blue mesh) for the AMPPNP and Mg^2+^ in AMPPNP-LolCDE (right). AMPPNP and Mg^2+^ are shown as stick (red) and spheres (green), respectively. The Walker A and signature motif are colored in blue and purple (right), respectively. **(B**) Cryo-EM maps of apo-LolCDE (left), RcsF-LolCDE (middle), and AMPPNP-LolCDE (right).
(PNG)

S7 Fig Sequence alignment and structural overlay of LolC with LolE. (**A**) Amino acid sequence alignment of LolC with LolE from *E*. *coli*. Alignments were made using Clustal Omega and colored in ESPript. (**B**) Overlay of PLDs of LolC (blue) with that of LolE (pink) from apo-LolCDE. **(C**) Overlay of TMDs of LolC (blue) with that of LolE (pink) from apo-LolCDE.
(PNG)

S8 Fig Structural overlay of LolCDE in different conformational states and the ATPase activity of LolCDE in different conditions. (**A**) Overlay of apo-LolCDE (blue) with RcsF-LolCDE (pink). **(B**) Overlay of AMPPNP-LolCDE (purple) with RcsF-LolCDE (pink). (**C**) Top view of TMDs of RcsF-LolCDE (left, pink) and AMPPNP-LolCDE (right, purple). (**D**) The ATPase activity of the nanodisc-embedded LolCDE andLolCD^E171Q^E. Data were analyzed by *t* tests. ****P < 0.0001. (**E**) The effects of the substrate RcsF (left) and/or LolA (right) on the ATPase activity of LolCDE in nanodisc. LolCDE were reconstituted with 1×, 5×, or 7× molar ratio of RcsF in nanodisc (left), and the right panel showing the ATPase activity of RcsF-LolCDE (molar ratio: RcsF:LolCDE = 7:1) in nanodisc that were incubated with 0.5×, 1×, 5×, or 10× molar excess of LolA. Data are analyzed by one-way ANOVA test. **P < 0.01; ***P < 0.01; ****P < 0.0001. Bars shown in (D) and (E) represent the averages of 3 replicates, with error bars showing the SD of ATPase activity. The source data for (D) and (E) are provided in S1 Data.
(PNG)

S9 Fig Structural comparison of RcsF-LolCDE and AMPPNP-LolCDE with the recently published structures. (**A**) Overlay of our RcsF-LolCDE (LolC, blue; LolE, pink; LolD, yellow or grey; RcsF, green) with lipoprotein-LolCDE (left, light purple, PDB: 7ARH) and lipoprotein-LolCDE (right, dark purple, PDB: 7MDX) give RMSD of 3.35 Å and 1.98 Å, respectively. Zoom-in view of the boxed region showing the alignment of RcsF (green) in our RcsF-LolCDE structure with lipoprotein in lipoprotein-LolCDE structure (left, light purple, PDB: 7ARH) and lipoprotein-LolCDE structure (right, dark purple, PDB: 7MDX). (**B**) Overlay of our AMPPNP-LolCDE (LolC, blue; LolE, pink; LolD, yellow or grey; AMPPNP, red; Mg^2+^, green) with AMPPNP-LolCDE (left, light purple, PDB: 7ARK) and vanadate-trapped LolCDE (right, dark purple, PDB: 7MDY) give RMSD of 3.30 Å and 3.05 Å, respectively. (**C**) Overlay of our apo-LolCDE (LolC, blue; LolE, pink; LolD, yellow or grey) with apo-LolCDE* (light purple, PDB: 7ARI) gives an RMSD of 5.9 Å.
(PNG)

S10 Fig Complementation assays in presence of L-arabinose. **(A** to **G**) Complementation assays (positive controls) for Figs 2E (**A**) and 3E (**B**) and 3H (**C**) and 4C (**D**) and 4F (**E**) and 5C (**F**) and 5E (**G**).
(PNG)

S11 Fig Protein stability evaluation of the wildtype *lolCDE* and *lolCDE* mutant proteins by size exclusion chromattography. (**A** to **F**) Size-exclusion chromatography profiles and Coomassie blue–stained SDS–PAGE analysis of the wild-type *lolCDE* and *lolCDE* mutant proteins for Figs 3D (**A**) and 3G (**B**) and 4B (**C**) and 4E (**D**) and 5B (**E**) and 5D (**F**).
(PNG)

S12 Fig Coomassie-stained SDS–PAGE analysis as loading control for the western blots of photo-crosslinking assays in Figs 3–5. **(A** to **F**) SDS-PAGE gels are the loading control of western blots for Figs 3D (**A**) and 3G (**B**) and 4B (**C**) and 4E (**D**) and 5B (**E**) and 5D (**F**).
(PNG)

S13 Fig Lipoprotein selectivity by LolCDE. **(A**) Zoom-in view of the hydrogen bond between Ser+2 of RcsF and Glu263 of LolC. The hydrogen bond is shown as dashed yellow line. (**B**) Cross-sectional side view of the hydrophobic surface of the V-shaped cavity of LolCDE showing the 2 negatively charged residues Glu263 (left) and Asp264 (right) in the substrate-binding cavity. Hydrophobic and hydrophilic regions are shown in blue and red, respectively. RcsF is colored in green, and Cys+1, Ser+2, and 3 acyl chains are shown as stick model. (**C**) Schematic structure of a bacterial lipoprotein (left), phospholipid (middle), and LPS (right). The first 2 amino acids of lipoproteins (Cys+1 and AA+2) and the negatively charged PO_4_^−^ groups of phospholipid and LPS are outlined in the red boxes, respectively. (**D**) Coomassie-stained SDS–PAGE gel assessing intermolecular disulfide bonds formation of LolC^L350C^DE^R263C^ and LolC^E255C^DE^S362C^. Note that the migration mass of LolC-LolE here is different from that in Fig 2B. The 2 pairs of intermolecular disulfide bonds in S13D Fig are LolC^L350C^-LolE^R263C^ and LolC^E255C^-LolE^S362C^ (both in the TMD regions of LolC and LolE); the intermolecular disulfide bond in Fig 2B is LolC^A106C^-E^S173C^ (in the PLDs of LolC and LolE). In any case, the LolC-LolE crosslinks disappeared upon addition of β-ME, and LolC and LolE appeared with the expected molecular masses on SDS-PAGE gels. (**E**) Ribbon diagram of LolA structure showing residue Trp70 (magenta spheres), which was substituted with *p*BPA. **(F**) Nanodisc-embedded RcsF-LolCDE or RcsF-LolC^L256*p*BPA^DE was incubated with LolA (W70*p*BPA) and ATP/Mg^2+^. In vitro lipoprotein transfer assays and photo-crosslinking showing that LolC^L256*p*BPA^DE is able to transfer RcsF to LolA (W70*p*BPA).
(PNG)

S14 Fig Multiple sequence alignment for LolC from different gram-negative bacterial strains. Amino acid sequence alignments of *E*. *coli*, *H*. *influenzae*, *V*. *cholerae*, *P*. *aeruginosa*, *S*. *enterica*, and *Y*. *pestis* for LolC. Abbreviations are as follows *E*. *coli*, *Escherichia coli*; *H*. *influenzae*, *Haemophilus influenzae*; *V*. *cholerae*, *Vibriocholerae*; *P*. *aeruginosa*, *Pseudomonas aeruginosa*; *S*. *enterica*, *Salmonella enterica; Y*. *pestis*, *Yersinia pestis*. Alignments were made using Clustal O and colored in ESPript. The mutations used in the study are labeled below the sequence.
(PNG)

S15 Fig Multiple sequence alignment for LolE from different gram-negative bacterial strains. Amino acid sequence alignments of *E*. *coli*, *H*. *influenzae*, *V*. *cholerae*, *P*. *aeruginosa*, *S*. *enterica*, and *Y*. *pestis* for LolE. Abbreviations are as follows *E*. *coli*, *Escherichia coli*; *H*. *influenzae*, *Haemophilus influenzae*; *V*. *cholerae*, *Vibriocholerae*; *P*. *aeruginosa*, *Pseudomonas aeruginosa*; *S*. *enterica*, *Salmonella enterica; Y*. *pestis*, *Yersinia pestis*. Alignments were made using Clustal O and colored in ESPript. The mutations used in the study are labeled below the sequence.
(PNG)

S16 Fig Mass spectrometry identification of LolE-LolC×RcsF crosslinking adducts. Mass spectrometry identification of RcsF peptide (IYTNAEELVGKPFR, top), LolC peptide (YPAGNITGWR, middle), and LolE peptide (LSALPSFVQGDAWR, bottom), verifying the LolE-LolC×RcsF crosslinking adducts in Fig 6B. The source data are provided in S1 Data.
(PNG)

S17 Fig Ribbon diagram of nucleotide-free (left) and ATP-bound (right) MacB structures. (PNG)

S1 Table Cryo-EM data collection, refinement, and validation statistics of apo-LolCDE, RcsF-LolCDE, and AMPPNP-LolCDE. (DOCX)

S1 Movie Conformation changes between RcsF-LolCDE and AMPPNP-LolCDE. The movie shows a morph between RcsF-LolCDE and AMPPNP-LolCDE.
(MP4)

S1 Data The numerical values underlying S8D and S8E and S13 Figs. (XLSX)

S1 Raw Images Uncropped images of SDS-PAGE gels and western blots of Figs 2B–2E and 2D and 2E and 3G-3I and 4B and 4C and 4E and 4F and 5B–5E and 6B and 6C and 6E and S1A–S1D and S11A–S11F and S12A–S12F and S13D and S13F. (PDF)

## Abbreviations

ABC: ATP-binding cassette
DDM: dodecyl maltoside
HRP: horseradish peroxidase
IM: inner membrane
LB: Luria broth
LPS: lipopolysaccharide
OM: outer membrane
*p*BPA: *p*-benzoyl-phenylalanine
PLD: periplasmic localized domain
Rcs: regulator of capsule synthesis
sgRNA: single guide RNA
TMD: transmembrane domain
β-ME: β-mercaptoethanol
