# Supplementary figures and images for: Cryo-EM structures of LolCDE reveal the molecular mechanism of bacterial lipoprotein sorting in Escherichia coli
Source: PLoS Biol. 2022 Oct 13;20(10):e3001823. doi: 10.1371/journal.pbio.3001823 (PMC9595528; doi:10.1371/journal.pbio.3001823)

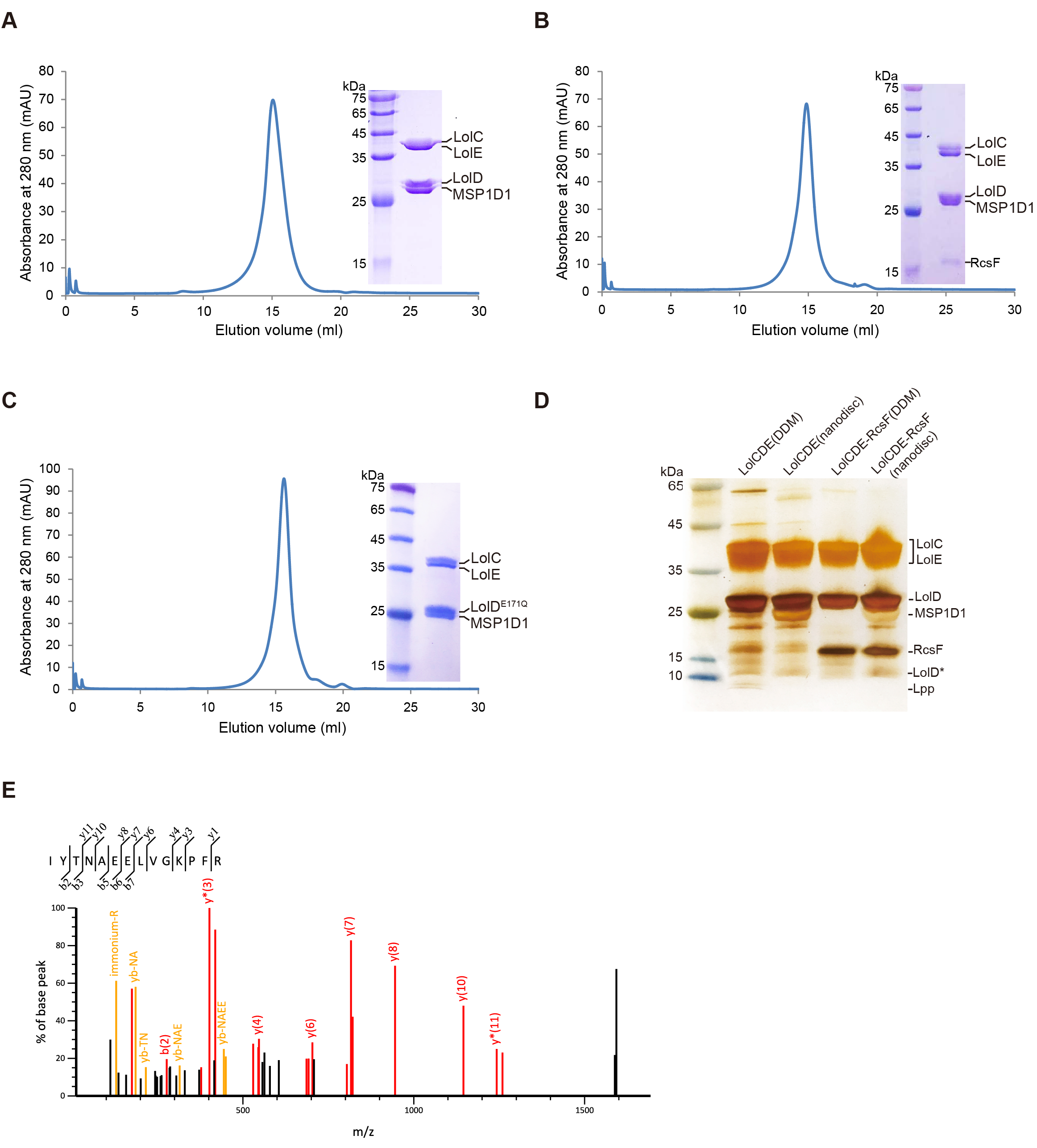

Supplement: S1 Fig — (A to C), Representative size-exclusion chromatography profiles and Coomassie blue–stained SDS–PAGE gel analysis of LolCDE (A), RcsF-LolCDE (B), and LolCDE171QE (C) in nanodisc. (D) Silver staining of LolCDE and RcsF-LolCDE samples that were used for structure determination. RcsF was copurified LolCDE; LolD* is the degraded LolD band. (E) Mass spectrometry identification of RcsF peptide (IYTNAEELVGKPFR). The experiments in (A to C) were repeated over 20 times. (PNG) [file pbio.3001823.s001.png]

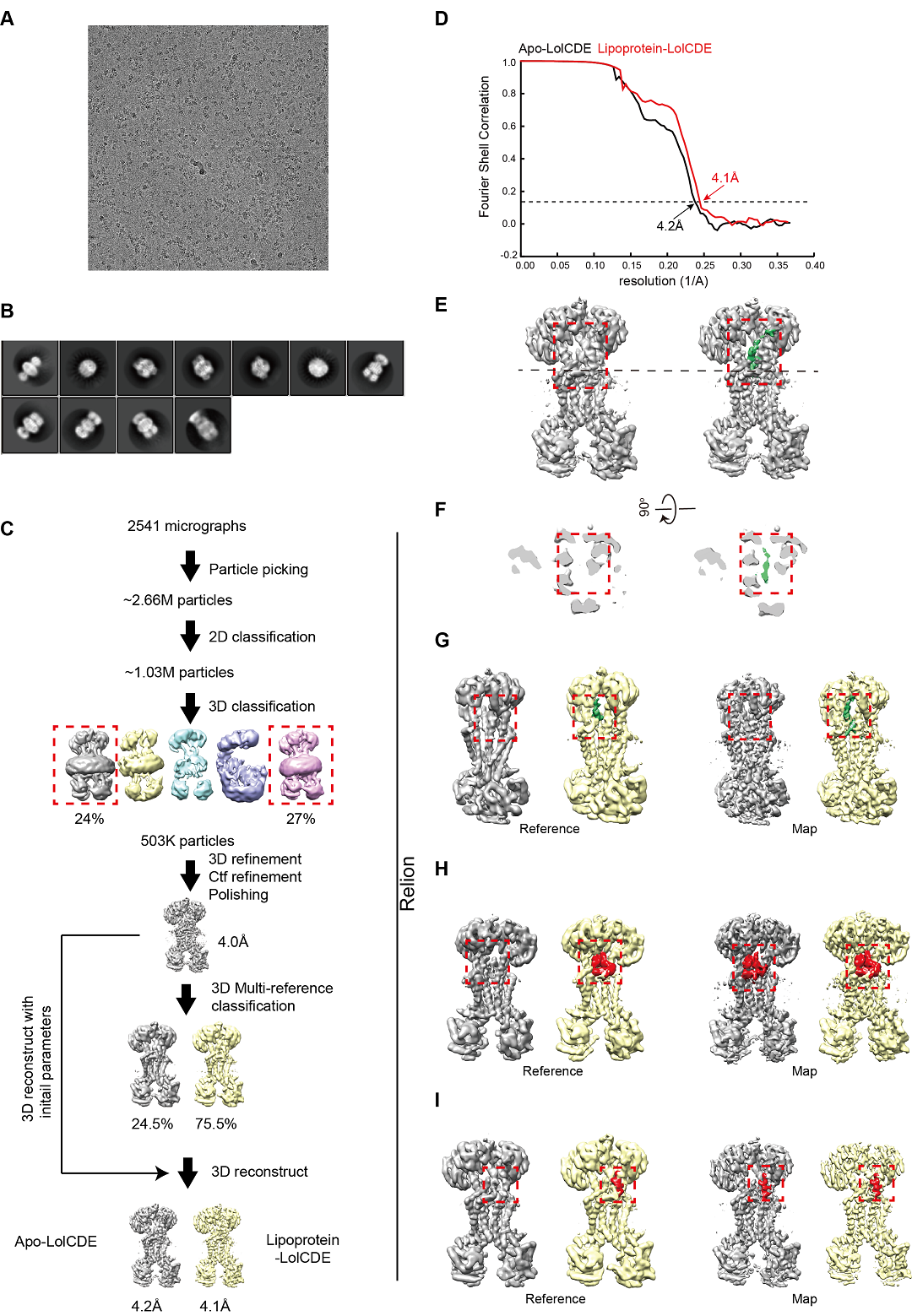

Supplement: S2 Fig — (A) Representative cryo-EM micrograph of LolCDE in nanodisc. (B) Selected 2D class averages of cryo-EM particle images. (C) Scheme of 3D classification and refinement of cryo-EM particle images. (D) Gold-standard Fourier shell correlation (FSC) curves of the final cryo-EM maps of LolCDE (4.2 Å) and lipoprotein-LolCDE (4.1 Å). The resolutions were determined at FSC = 0.143. (E) The cryo-EM maps of apo-LolCDE (left) and lipoprotein-LolCDE (right). The lipoprotein densities are colored in green. (F) Top-down view of the slice through the cryo-EM map of apo-LolCDE (left) and lipoprotein-LolCDE (right), as indicated by the black dotted line in (E). The extra densities of lipoprotein (green) were only observed in the slice through the cryo-EM map of lipoprotein-LolCDE. (G) Verification classification with 2 references that one excluded RcsF densities and the other excluded the densities of 3 acyl chains but not the RcsF proteinaceous parts, showing that extra densities of the proteinaceous parts and 3 acyl chains both existed in 1 map. (H) Verification classification with 2 references that one excluded the densities of the LoopLolE, showing that the densities of LoopLolE lacked in reference still existed in the final maps. (I) The verification classification with 2 references that one excluded the densities of partial TM2LolE, showing the densities of partial TM2LolE lacked in reference still existed in the final maps. (PNG) [file pbio.3001823.s002.png]

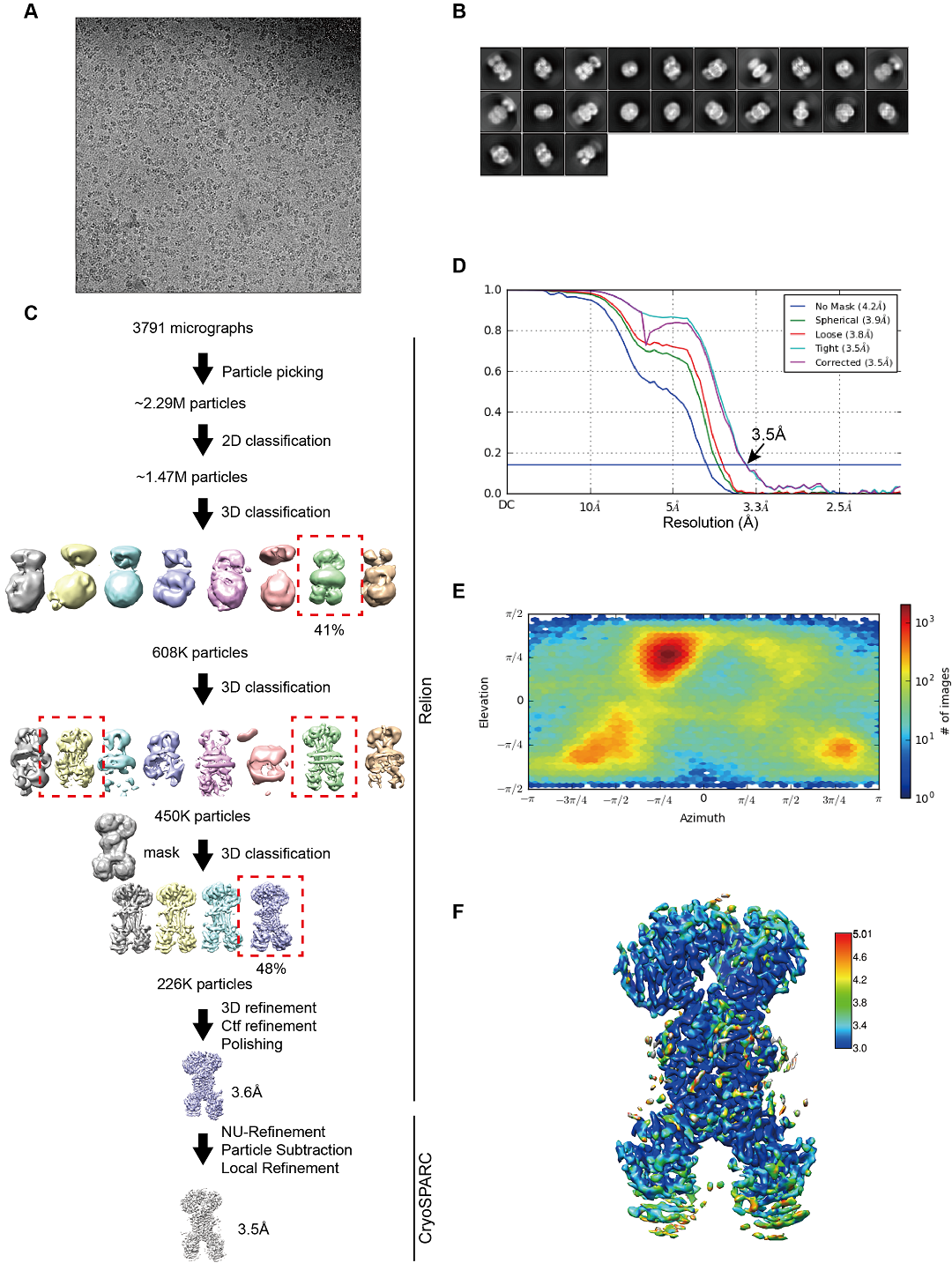

Supplement: S3 Fig — (A) Representative cryo-EM micrograph of RcsF-LolCDE in nanodisc. (B) Selected 2D class averages of cryo-EM particle images. (C) Scheme of 3D classification and refinement of cryo-EM particle images. (D) Gold-standard FSC curves calculated with different masks in cryoSPARC. The resolutions were determined at FSC = 0.143 (horizontal blue line). The final corrected mask gave an overall resolution of 3.5 Å. (E) Distribution of orientations over azimuth and elevation angles for particles included in the calculation of the final map. (F) Cryo-EM map of RcsF-LolCDE colored by local resolution. (PNG) [file pbio.3001823.s003.png]

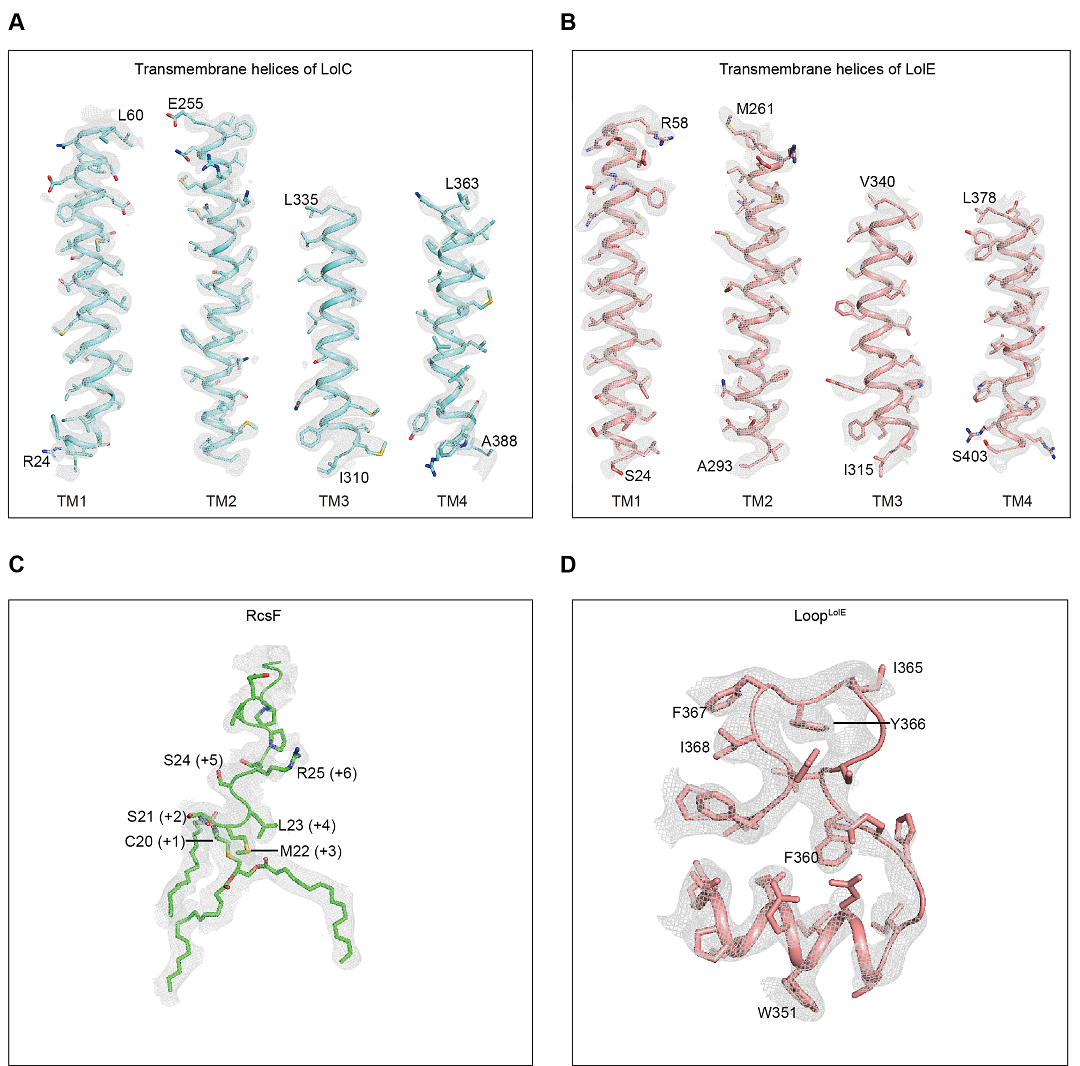

Supplement: S4 Fig — (A to D) Cryo-EM densities superimposed with the atomic model for TMs of LolC (A), TMs of LolE (B), RcsF(C), and LoopLolE (D), respectively. (PNG) [file pbio.3001823.s004.png]

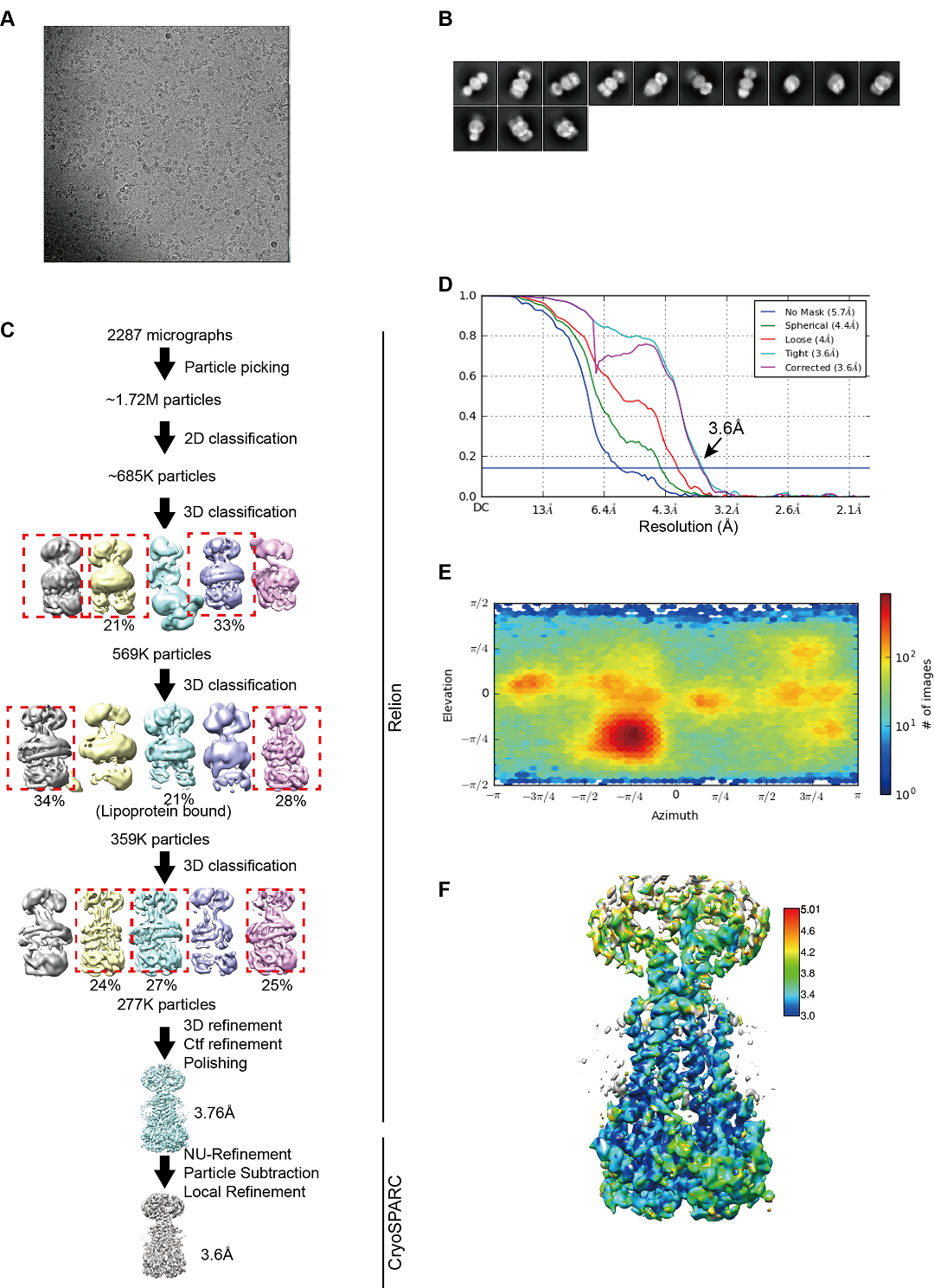

Supplement: S5 Fig — (A) Representative cryo-EM micrograph of AMPPNP-LolCDE in nanodisc. (B) Selected 2D class averages of cryo-EM particle images. (C) Scheme of 3D classification and refinement of cryo-EM particle images. (D) Gold-standard FSC curves calculated with different masks in cryoSPARC. The resolutions were determined at FSC = 0.143 (horizontal blue line). The final corrected mask gave an overall resolution of 3.6 Å. (E) Distribution of orientations over azimuth and elevation angles for particles included in the calculation of the final map. (F) Cryo-EM map of AMPPNP-LolCDE colored by local resolution. (PNG) [file pbio.3001823.s005.png]

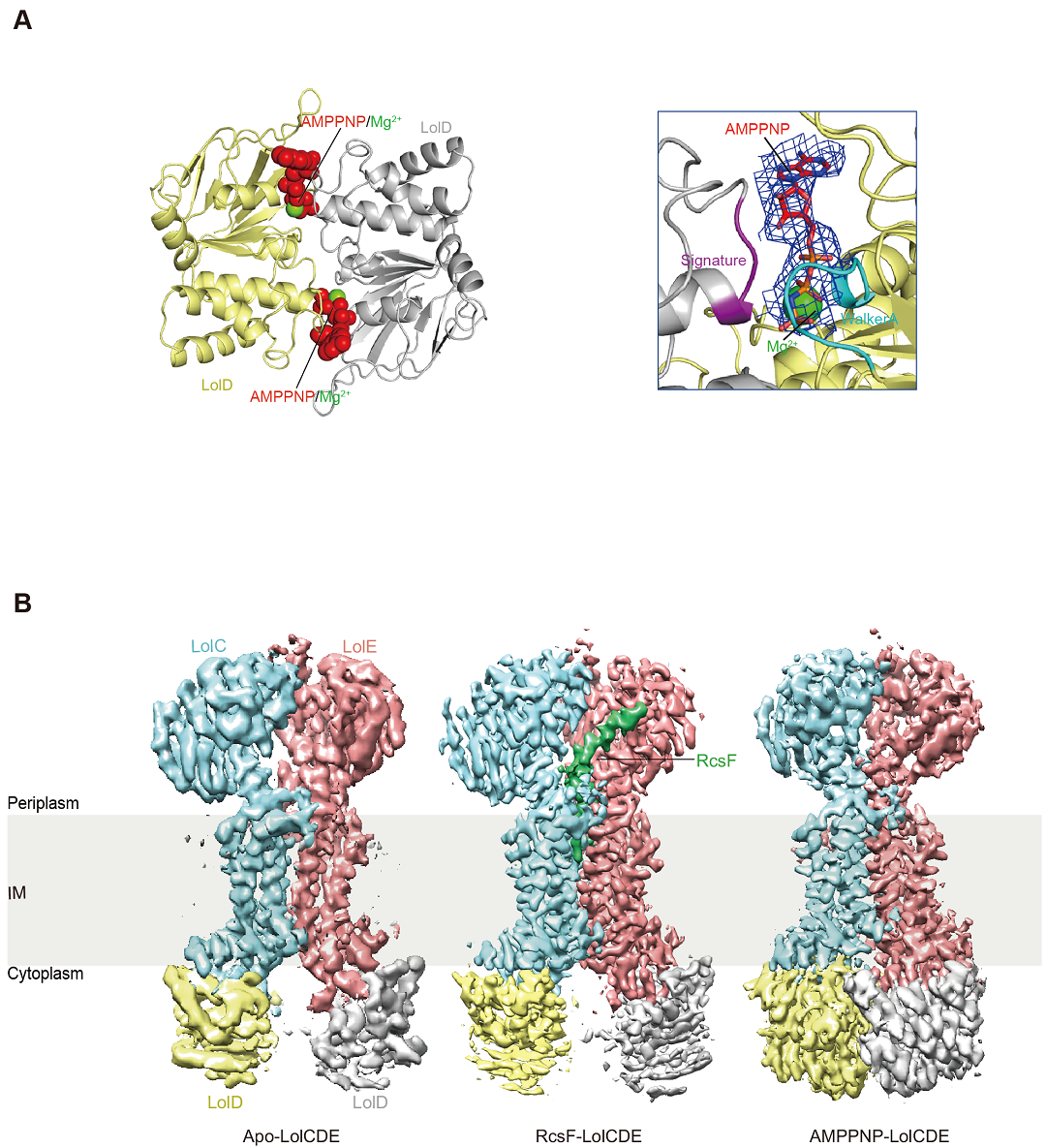

Supplement: S6 Fig — (A) Location of the 2 AMPPNP (red spheres) and Mg2+ (green spheres) molecules clamped between the 2 LolD subunits (left). Cryo-EM densities (deep blue mesh) for the AMPPNP and Mg2+ in AMPPNP-LolCDE (right). AMPPNP and Mg2+ are shown as stick (red) and spheres (green), respectively. The Walker A and signature motif are colored in blue and purple (right), respectively. (B) Cryo-EM maps of apo-LolCDE (left), RcsF-LolCDE (middle), and AMPPNP-LolCDE (right). (PNG) [file pbio.3001823.s006.png]

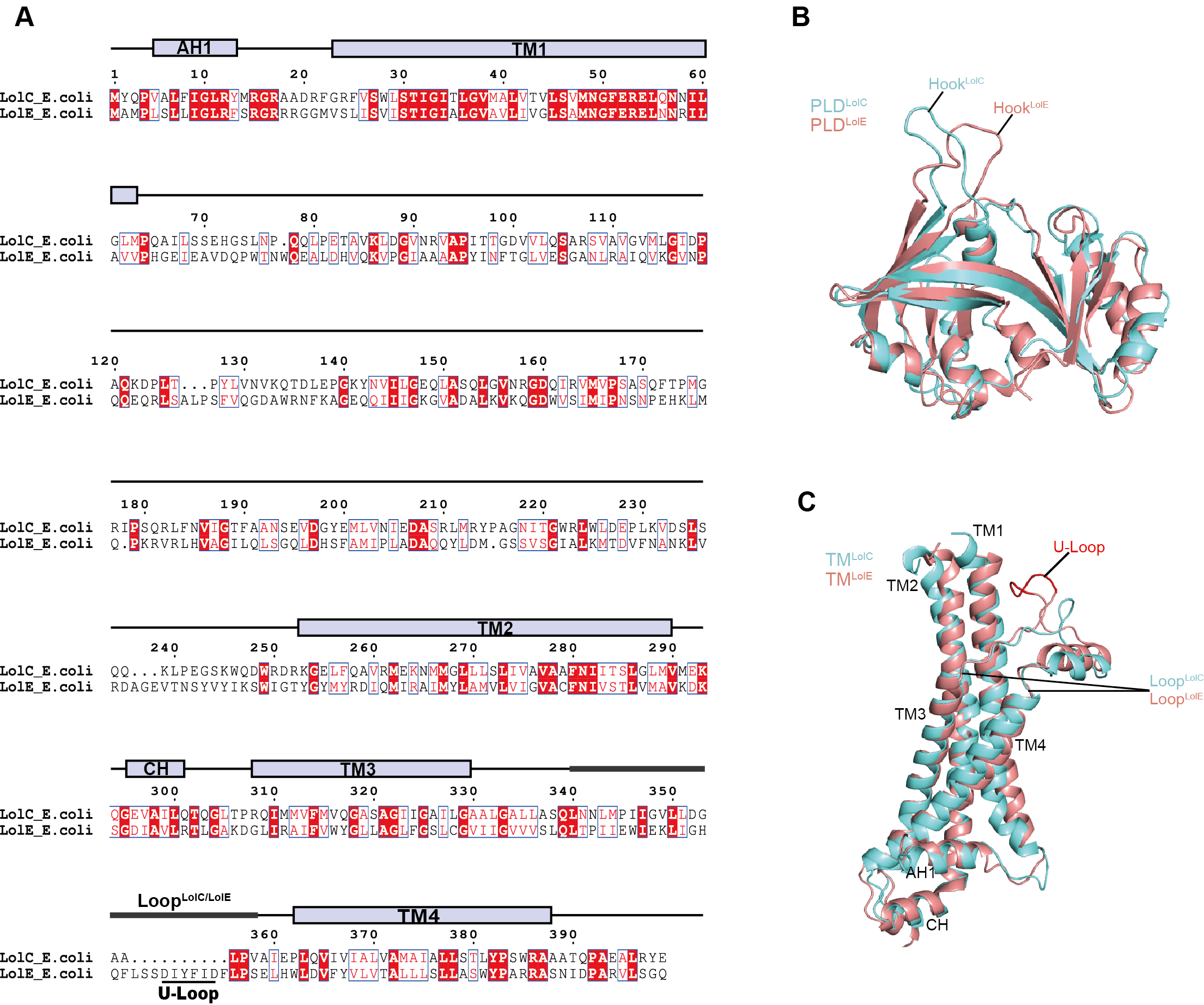

Supplement: S7 Fig — (A) Amino acid sequence alignment of LolC with LolE from E. coli. Alignments were made using Clustal Omega and colored in ESPript. (B) Overlay of PLDs of LolC (blue) with that of LolE (pink) from apo-LolCDE. (C) Overlay of TMDs of LolC (blue) with that of LolE (pink) from apo-LolCDE. (PNG) [file pbio.3001823.s007.png]

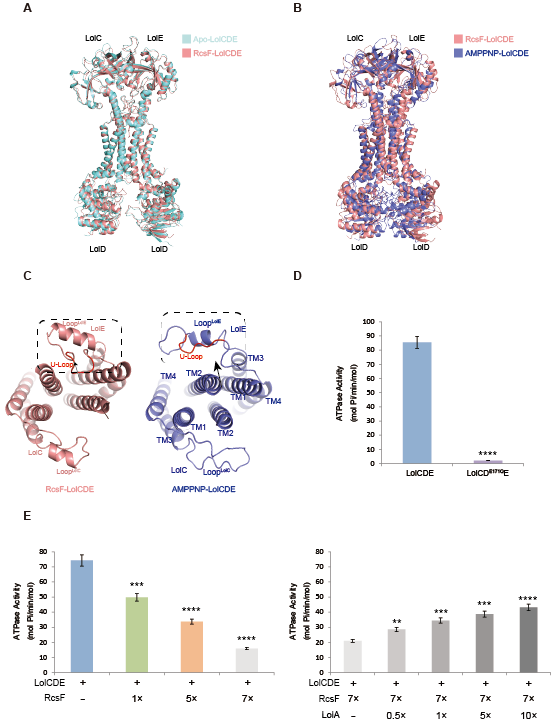

Supplement: S8 Fig — (A) Overlay of apo-LolCDE (blue) with RcsF-LolCDE (pink). (B) Overlay of AMPPNP-LolCDE (purple) with RcsF-LolCDE (pink). (C) Top view of TMDs of RcsF-LolCDE (left, pink) and AMPPNP-LolCDE (right, purple). (D) The ATPase activity of the nanodisc-embedded LolCDE andLolCDE171QE. Data were analyzed by t tests. ****P < 0.0001. (E) The effects of the substrate RcsF (left) and/or LolA (right) on the ATPase activity of LolCDE in nanodisc. LolCDE were reconstituted with 1×, 5×, or 7× molar ratio of RcsF in nanodisc (left), and the right panel showing the ATPase activity of RcsF-LolCDE (molar ratio: RcsF:LolCDE = 7:1) in nanodisc that were incubated with 0.5×, 1×, 5×, or 10× molar excess of LolA. Data are analyzed by one-way ANOVA test. **P < 0.01; ***P < 0.01; ****P < 0.0001. Bars shown in (D) and (E) represent the averages of 3 replicates, with error bars showing the SD of ATPase activity. The source data for (D) and (E) are provided in S1 Data. (PNG) [file pbio.3001823.s008.png]

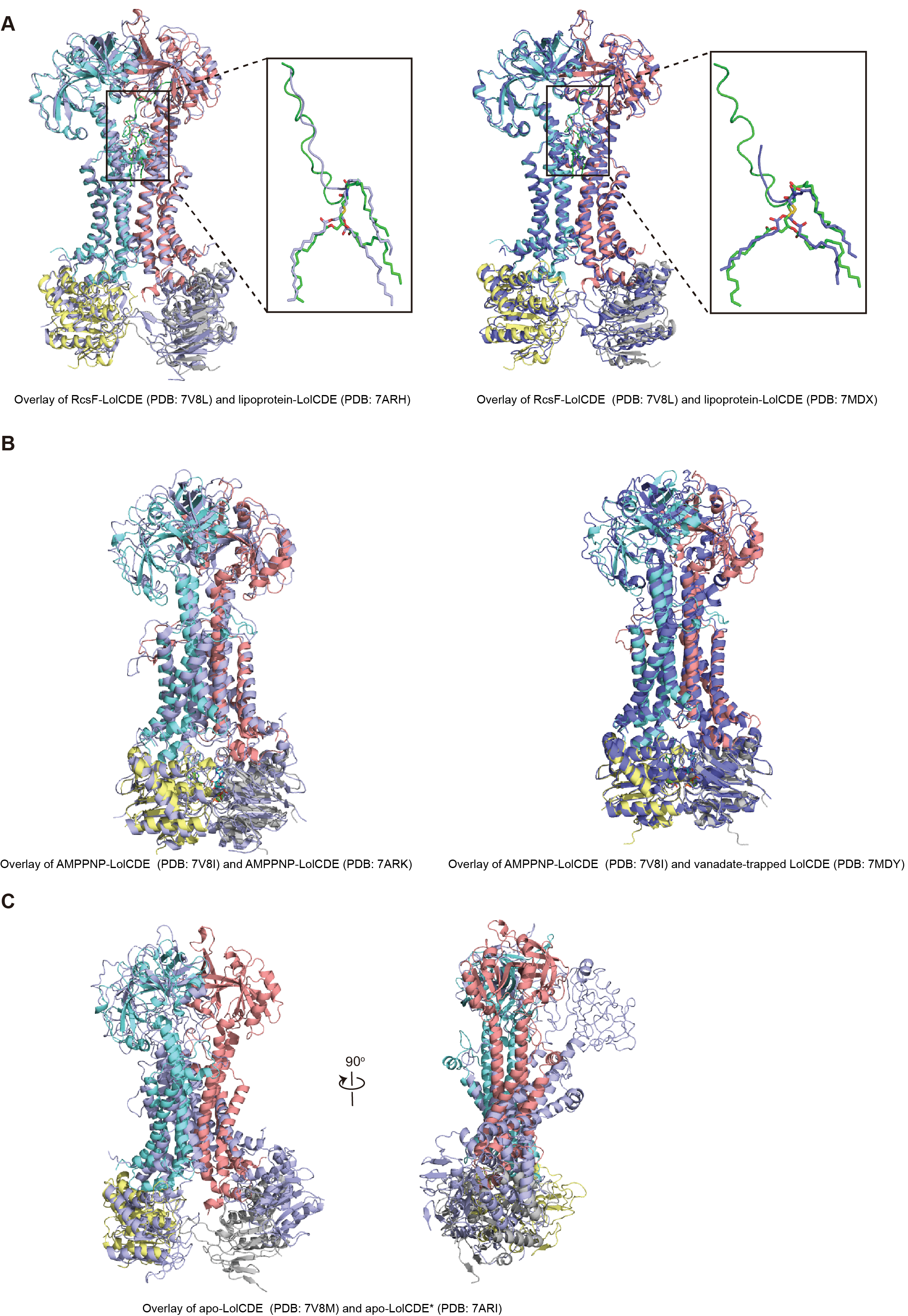

Supplement: S9 Fig — (A) Overlay of our RcsF-LolCDE (LolC, blue; LolE, pink; LolD, yellow or grey; RcsF, green) with lipoprotein-LolCDE (left, light purple, PDB: 7ARH) and lipoprotein-LolCDE (right, dark purple, PDB: 7MDX) give RMSD of 3.35 Å and 1.98 Å, respectively. Zoom-in view of the boxed region showing the alignment of RcsF (green) in our RcsF-LolCDE structure with lipoprotein in lipoprotein-LolCDE structure (left, light purple, PDB: 7ARH) and lipoprotein-LolCDE structure (right, dark purple, PDB: 7MDX). (B) Overlay of our AMPPNP-LolCDE (LolC, blue; LolE, pink; LolD, yellow or grey; AMPPNP, red; Mg2+, green) with AMPPNP-LolCDE (left, light purple, PDB: 7ARK) and vanadate-trapped LolCDE (right, dark purple, PDB: 7MDY) give RMSD of 3.30 Å and 3.05 Å, respectively. (C) Overlay of our apo-LolCDE (LolC, blue; LolE, pink; LolD, yellow or grey) with apo-LolCDE* (light purple, PDB: 7ARI) gives an RMSD of 5.9 Å. (PNG) [file pbio.3001823.s009.png]

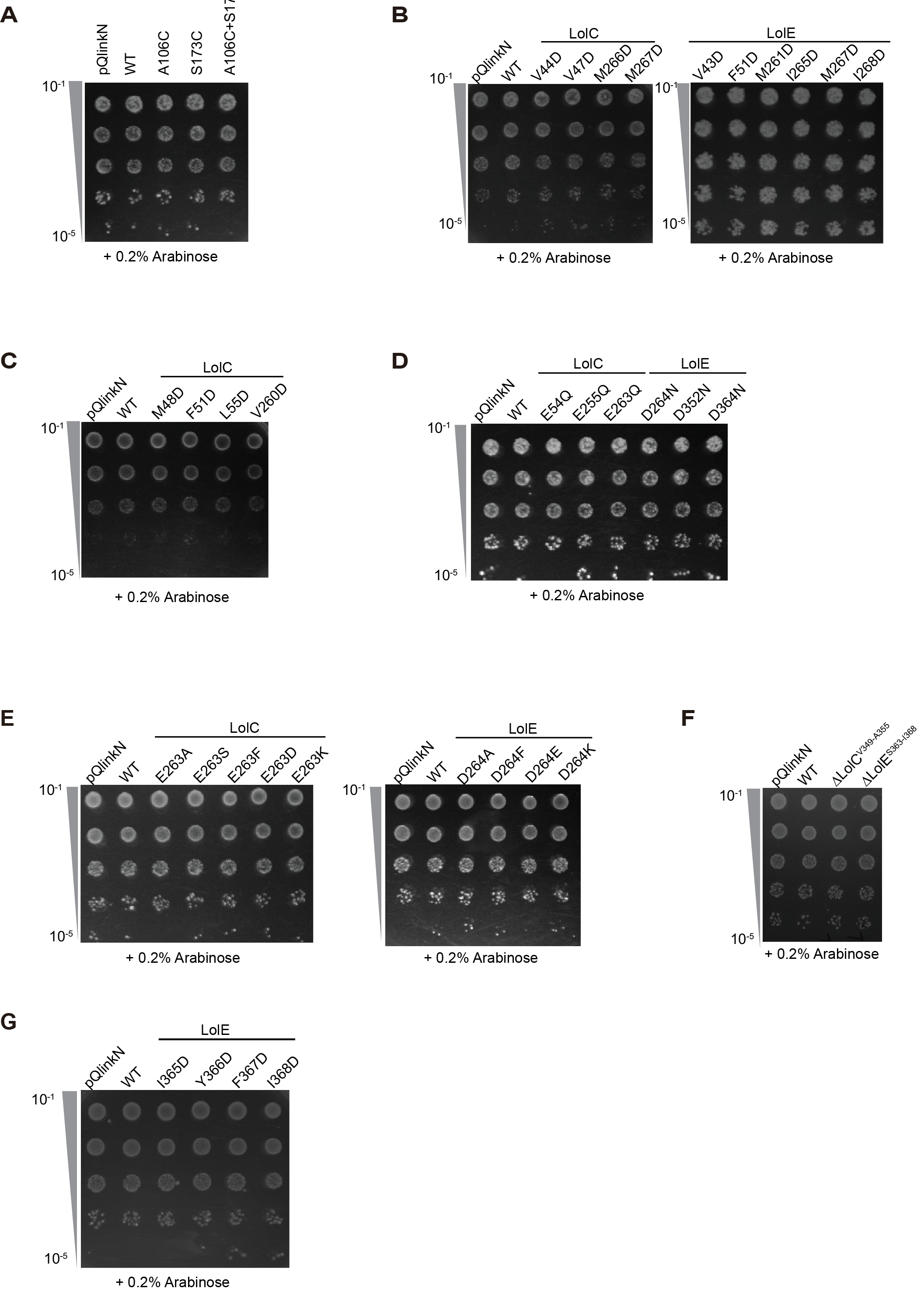

Supplement: S10 Fig — (A to G) Complementation assays (positive controls) for Figs 2E (A) and 3E (B) and 3H (C) and 4C (D) and 4F (E) and 5C (F) and 5E (G). (PNG) [file pbio.3001823.s010.png]

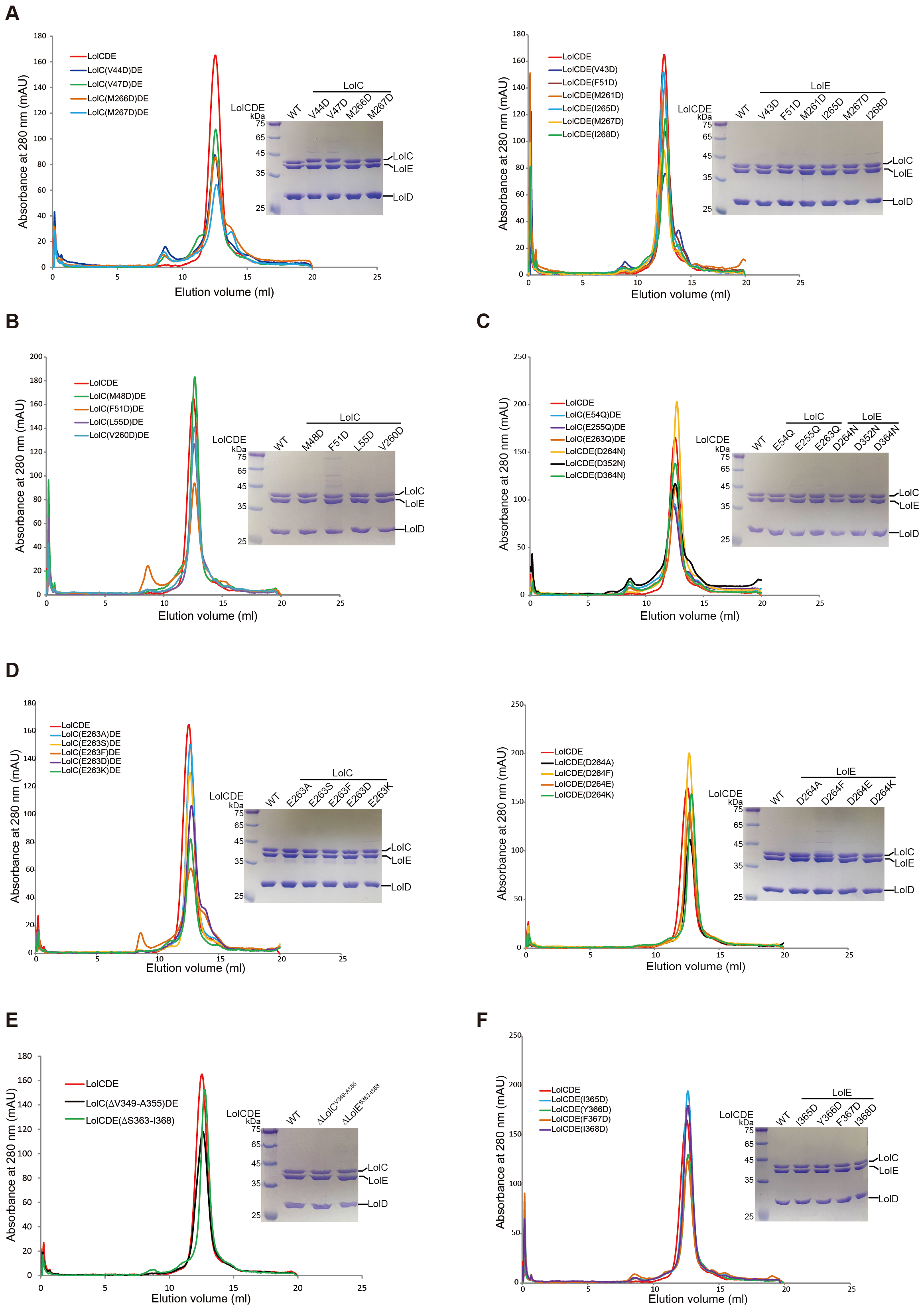

Supplement: S11 Fig — (A to F) Size-exclusion chromatography profiles and Coomassie blue–stained SDS–PAGE analysis of the wild-type lolCDE and lolCDE mutant proteins for Figs 3D (A) and 3G (B) and 4B (C) and 4E (D) and 5B (E) and 5D (F). (PNG) [file pbio.3001823.s011.png]

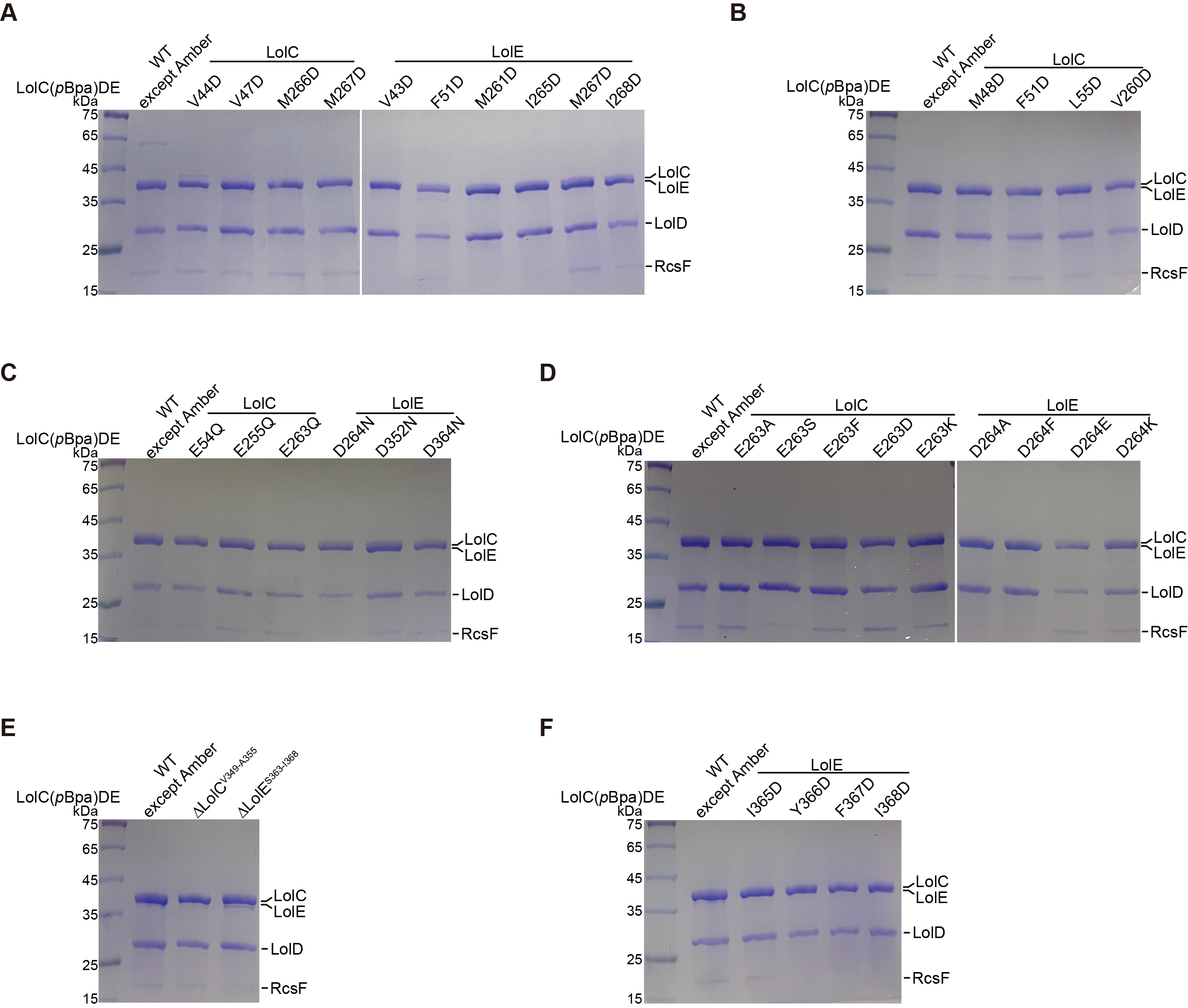

Supplement: S12 Fig — (A to F) SDS-PAGE gels are the loading control of western blots for Figs 3D (A) and 3G (B) and 4B (C) and 4E (D) and 5B (E) and 5D (F). (PNG) [file pbio.3001823.s012.png]

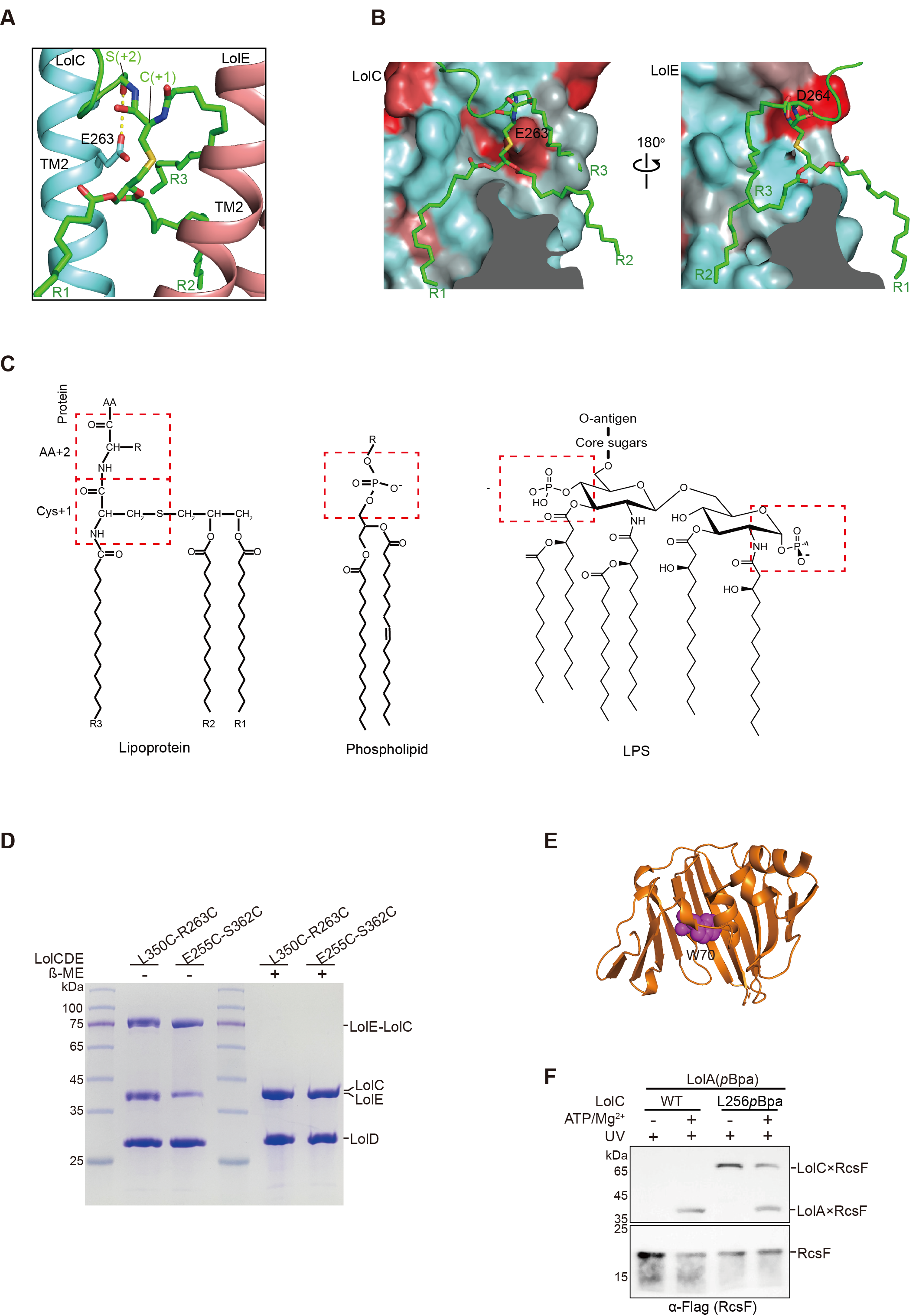

Supplement: S13 Fig — (A) Zoom-in view of the hydrogen bond between Ser+2 of RcsF and Glu263 of LolC. The hydrogen bond is shown as dashed yellow line. (B) Cross-sectional side view of the hydrophobic surface of the V-shaped cavity of LolCDE showing the 2 negatively charged residues Glu263 (left) and Asp264 (right) in the substrate-binding cavity. Hydrophobic and hydrophilic regions are shown in blue and red, respectively. RcsF is colored in green, and Cys+1, Ser+2, and 3 acyl chains are shown as stick model. (C) Schematic structure of a bacterial lipoprotein (left), phospholipid (middle), and LPS (right). The first 2 amino acids of lipoproteins (Cys+1 and AA+2) and the negatively charged PO4− groups of phospholipid and LPS are outlined in the red boxes, respectively. (D) Coomassie-stained SDS–PAGE gel assessing intermolecular disulfide bonds formation of LolCL350CDER263C and LolCE255CDES362C. Note that the migration mass of LolC-LolE here is different from that in Fig 2B. The 2 pairs of intermolecular disulfide bonds in S13D Fig are LolCL350C-LolER263C and LolCE255C-LolES362C (both in the TMD regions of LolC and LolE); the intermolecular disulfide bond in Fig 2B is LolCA106C-ES173C (in the PLDs of LolC and LolE). In any case, the LolC-LolE crosslinks disappeared upon addition of β-ME, and LolC and LolE appeared with the expected molecular masses on SDS-PAGE gels. (E) Ribbon diagram of LolA structure showing residue Trp70 (magenta spheres), which was substituted with pBPA. (F) Nanodisc-embedded RcsF-LolCDE or RcsF-LolCL256pBPADE was incubated with LolA (W70pBPA) and ATP/Mg2+. In vitro lipoprotein transfer assays and photo-crosslinking showing that LolCL256pBPADE is able to transfer RcsF to LolA (W70pBPA). (PNG) [file pbio.3001823.s013.png]

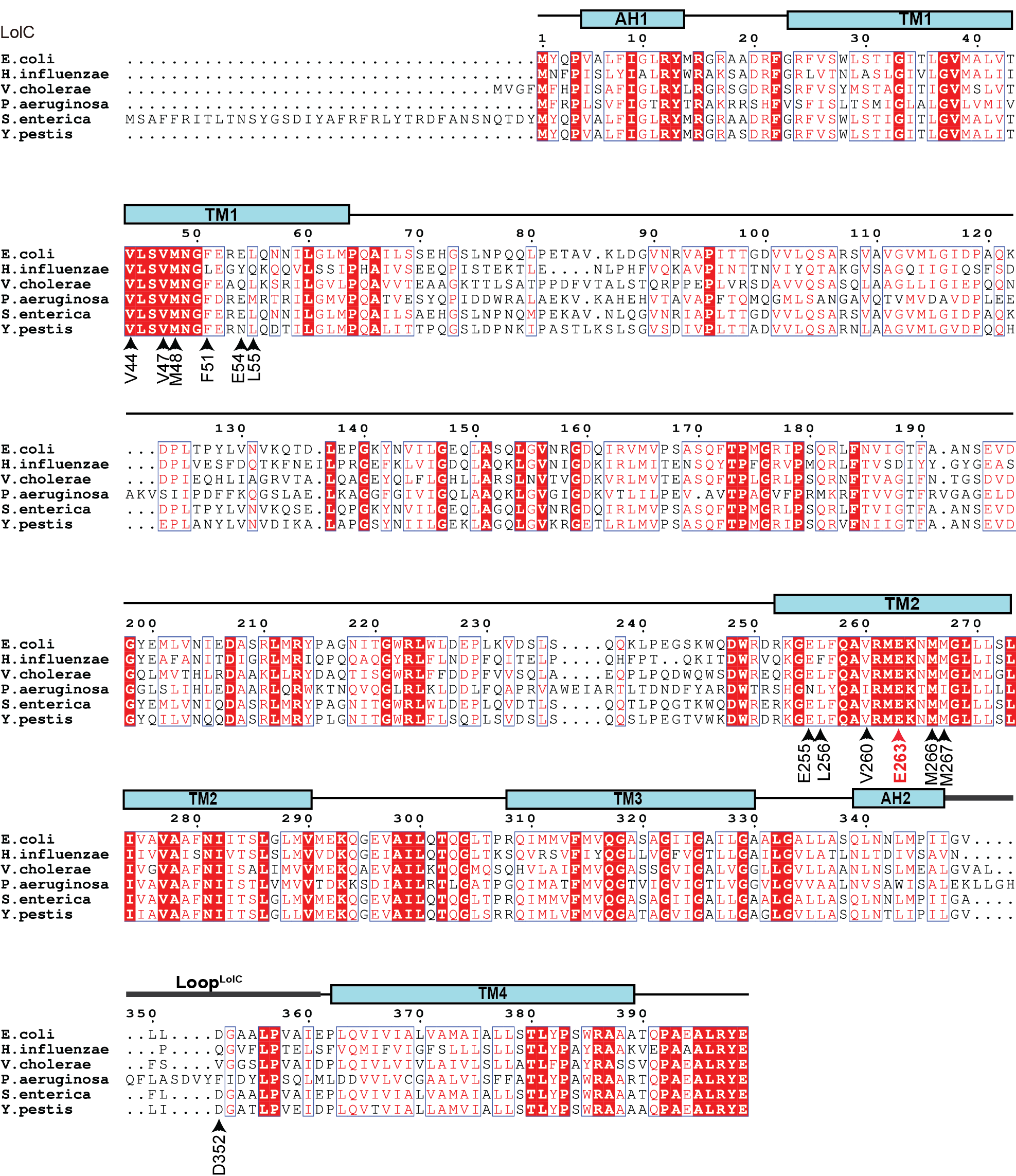

Supplement: S14 Fig — Amino acid sequence alignments of E. coli, H. influenzae, V. cholerae, P. aeruginosa, S. enterica, and Y. pestis for LolC. Abbreviations are as follows E. coli, Escherichia coli; H. influenzae, Haemophilus influenzae; V. cholerae, Vibriocholerae; P. aeruginosa, Pseudomonas aeruginosa; S. enterica, Salmonella enterica; Y. pestis, Yersinia pestis. Alignments were made using Clustal O and colored in ESPript. The mutations used in the study are labeled below the sequence. (PNG) [file pbio.3001823.s014.png]

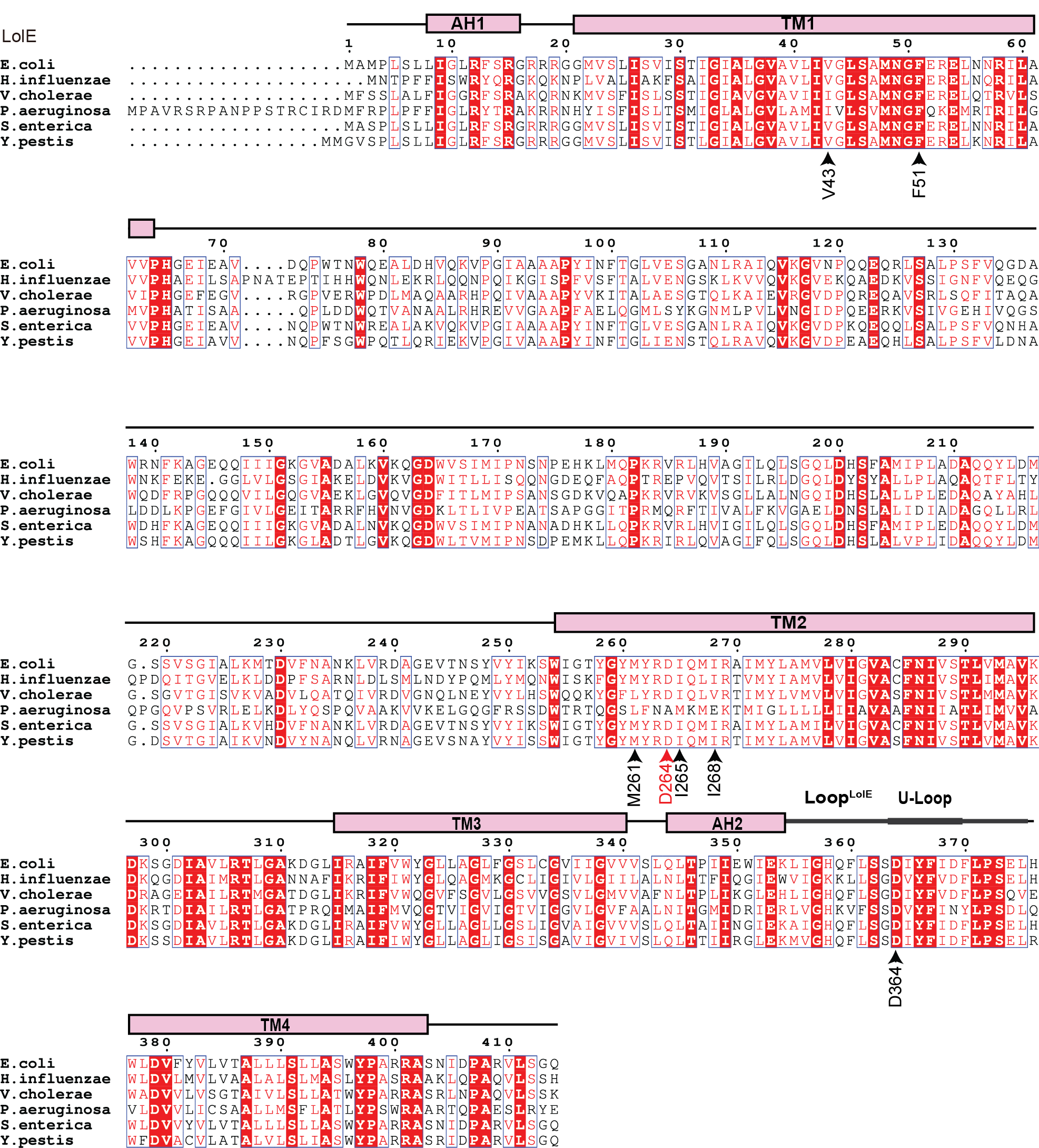

Supplement: S15 Fig — Amino acid sequence alignments of E. coli, H. influenzae, V. cholerae, P. aeruginosa, S. enterica, and Y. pestis for LolE. Abbreviations are as follows E. coli, Escherichia coli; H. influenzae, Haemophilus influenzae; V. cholerae, Vibriocholerae; P. aeruginosa, Pseudomonas aeruginosa; S. enterica, Salmonella enterica; Y. pestis, Yersinia pestis. Alignments were made using Clustal O and colored in ESPript. The mutations used in the study are labeled below the sequence. (PNG) [file pbio.3001823.s015.png]

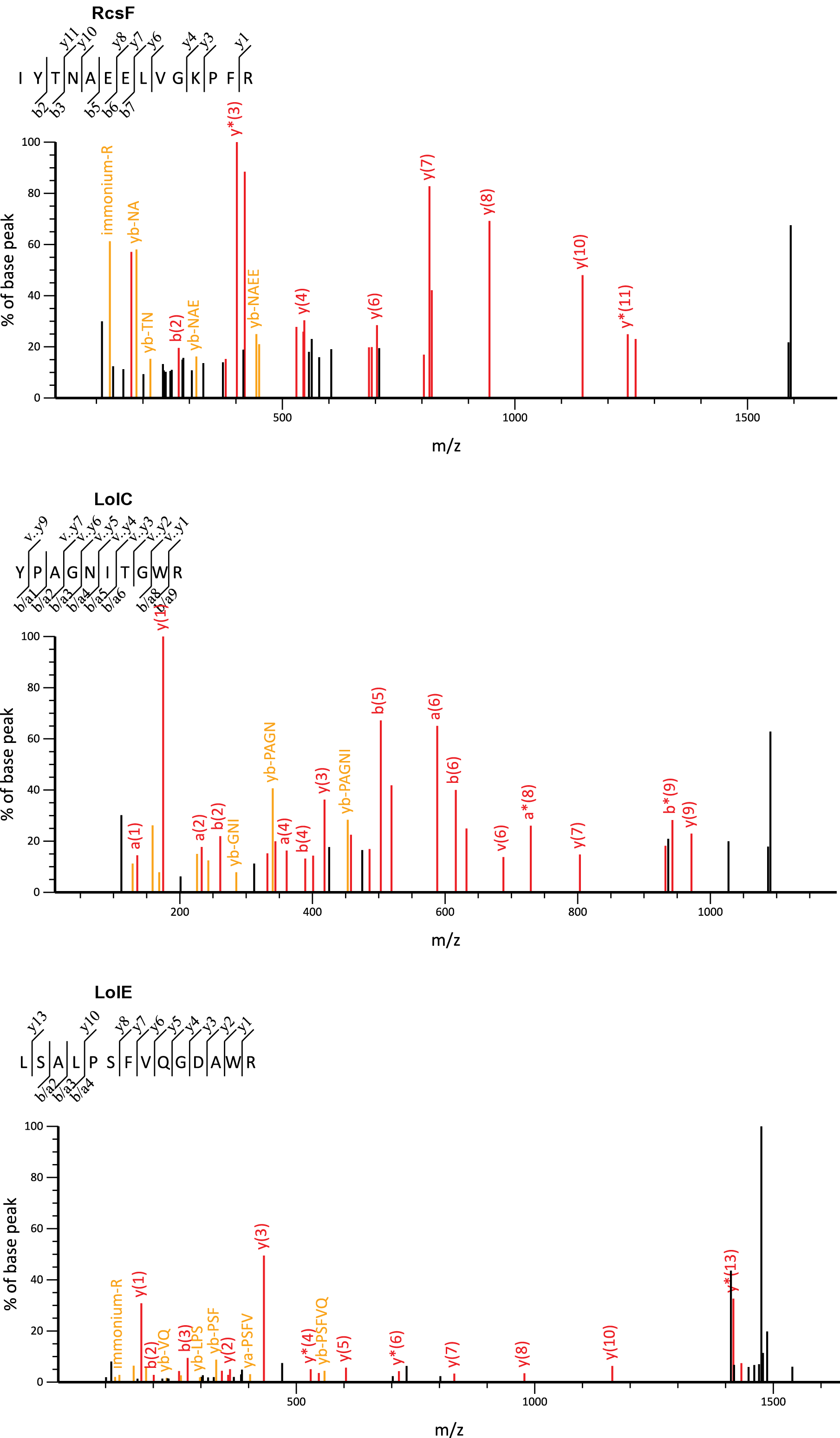

Supplement: S16 Fig — Mass spectrometry identification of RcsF peptide (IYTNAEELVGKPFR, top), LolC peptide (YPAGNITGWR, middle), and LolE peptide (LSALPSFVQGDAWR, bottom), verifying the LolE-LolC×RcsF crosslinking adducts in Fig 6B. The source data are provided in S1 Data. (PNG) [file pbio.3001823.s016.png]

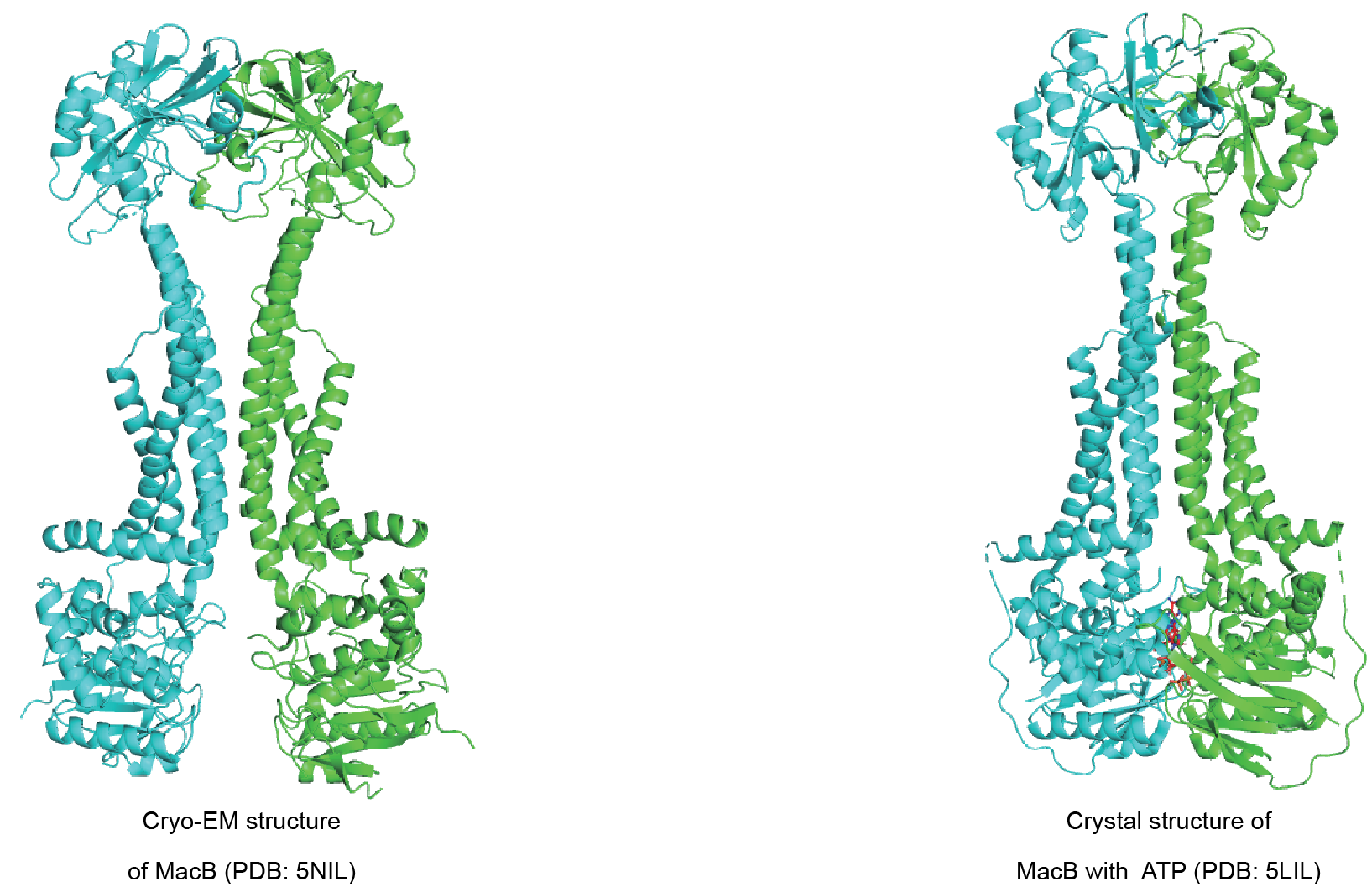

Supplement: S17 Fig — (PNG) [file pbio.3001823.s017.png]
