# Supplementary material for: Cryo-EM structures of LolCDE reveal the molecular mechanism of bacterial lipoprotein sorting in Escherichia coli
Source: PLoS Biol. 2022 Oct 13;20(10):e3001823. doi: 10.1371/journal.pbio.3001823 (PMC9595528; doi:10.1371/journal.pbio.3001823)
Supplement: S1 Table — (DOCX) [file pbio.3001823.s018.docx]

S1 Table. Cryo-EM data collection, refinement, and validation statistics of apo-LolCDE, RcsF-LolCDE and AMPPNP-LolCDE

|  | Apo-LolCDE | RcsF-LolCDE | AMPPNP-LolCDE |
| --- | --- | --- | --- |
| **Data collection and processing** |  |  |  |
| Magnification | 130,000 | 130,000 | 22,000 |
| Voltage (kV) | 300 | 300 | 200 |
| Electron exposure (e^–^/Å^2^) | 60 | 60 | 50 |
| Defocus range (μm) | -1.8~-2.2 | -1.8~-2.2 | -1.8~-2.2 |
| Pixel size (Å) | 1.36 | 1.04 | 1.00 |
| Symmetry imposed | C1 | C1 | C1 |
| Initial particle images (no.) | 2,666,570 | 2,293,045 | 1,109,045 |
| Final particle images (no.) | 135,391 | 225,933 | 277,296 |
| Map resolution (Å) | 4.2 | 3.5 | 3.6 |
| FSC threshold | 0.143 | 0.143 | 0.143 |
| Map resolution range (Å) | 3.9~6.5 | 3.1~5.3 | 3.2~7.0 |
|  |  |  |  |
| **Refinement** |  |  |  |
| Initial model | RcsF-LolCDE | 5NAA/5UDF/2PCJ | RcsF-LolCDE |
| Map sharpening *B* factor (Å^2^) | -108 | -124 | -119 |
| Model composition |  |  |  |
| Non-hydrogen atoms | 9,338 | 9,504 | 9381 |
| Protein residues | 1,263 | 1,277 | 1,260 |
| Ligands  Mg^2+^ | -  - | 1  - | 2  2 |
| *B*-factors (Å^2^) |  |  |  |
| Protein | 55.2 | 55.3 | 189.7 |
| Ligand | - | 20 | 37 |
| R.m.s. deviations |  |  |  |
| Bond lengths (Å) | 0.003 | 0.006 | 0.003 |
| Bond angles (º) | 0.715 | 0.876 | 0.720 |
| Validation |  |  |  |
| MolProbity score | 1.98 | 1.84 | 1.84 |
| Clashscore | 14.91 | 12.96 | 11.67 |
| Poor rotamers (%) | 0 | 0.1 | 0 |
| Ramachandran Plot |  |  |  |
| Favored (%) | 95.70 | 96.61 | 96.25 |
| Allowed (%) | 4.30 | 3.31 | 3.75 |
| Disallowed (%) | 0 | 0.08 | 0 |
