# Supplementary material for: Cryo-EM structures of LolCDE reveal the molecular mechanism of bacterial lipoprotein sorting in Escherichia coli
Source: PLoS Biol. 2022 Oct 13;20(10):e3001823. doi: 10.1371/journal.pbio.3001823 (PMC9595528; doi:10.1371/journal.pbio.3001823)
Supplement: S1 Raw Images — (PDF) [file pbio.3001823.s021.pdf]

Fig 2. Verification of the apo-LolCDE structure

B

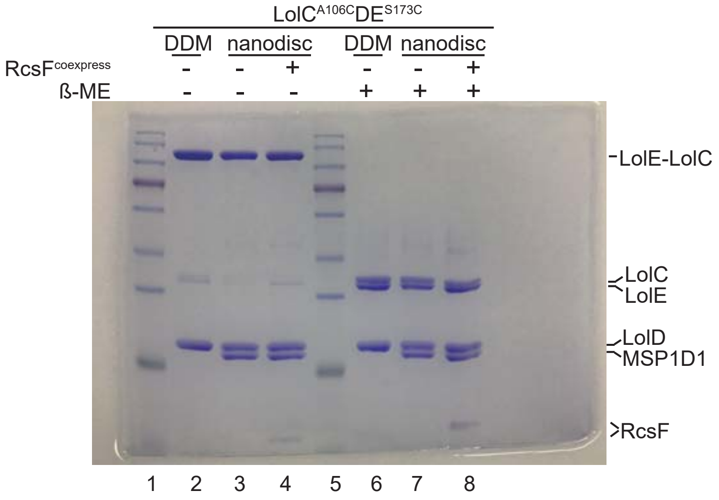

Image captured by camera

C

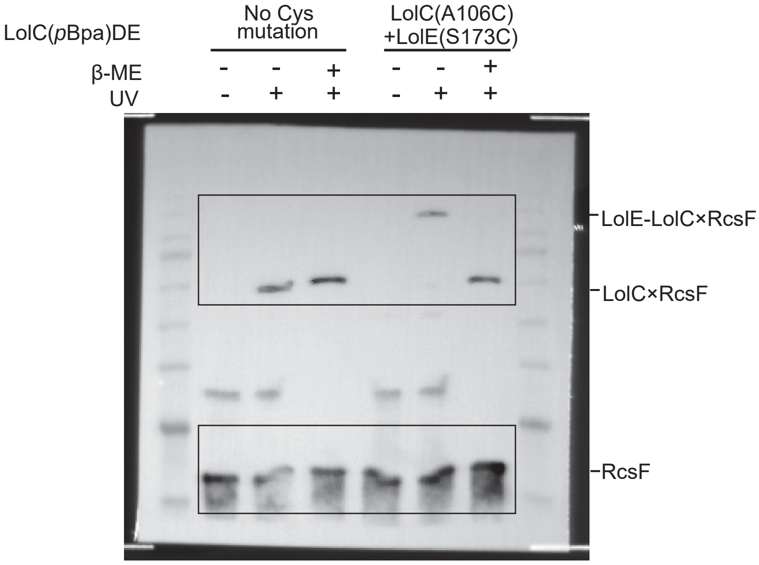

Image captured by ChemiScope3500 Mini (CLNX)

D

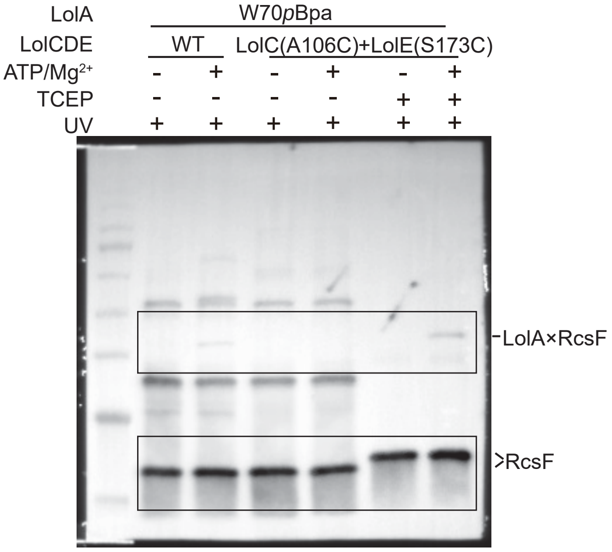

Image captured by ChemiScope3500 Mini (CLNX)

E

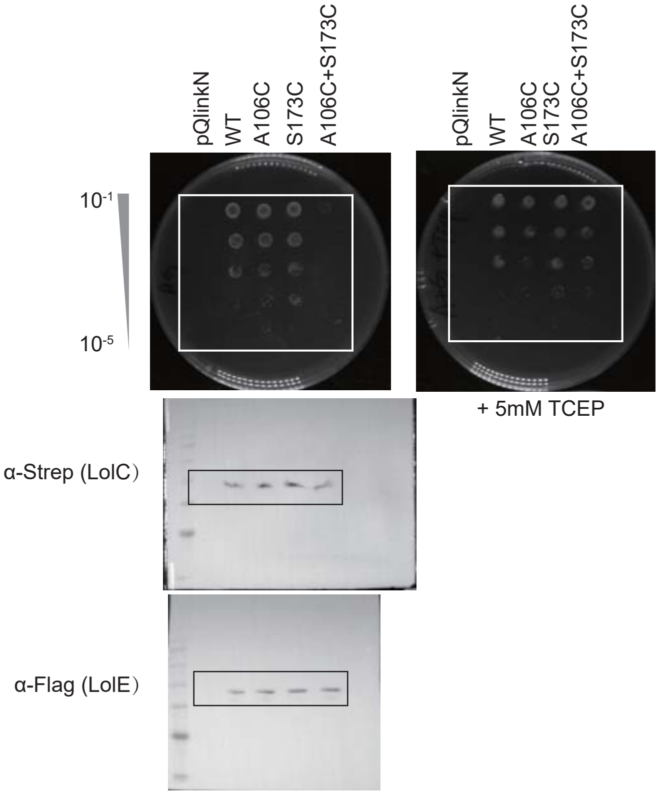

Image captured by GenoSens and ChemiScope3500 Mini (CLNX)

Fig 3. The bipartite binding mode between RcsF and LolCDE

D

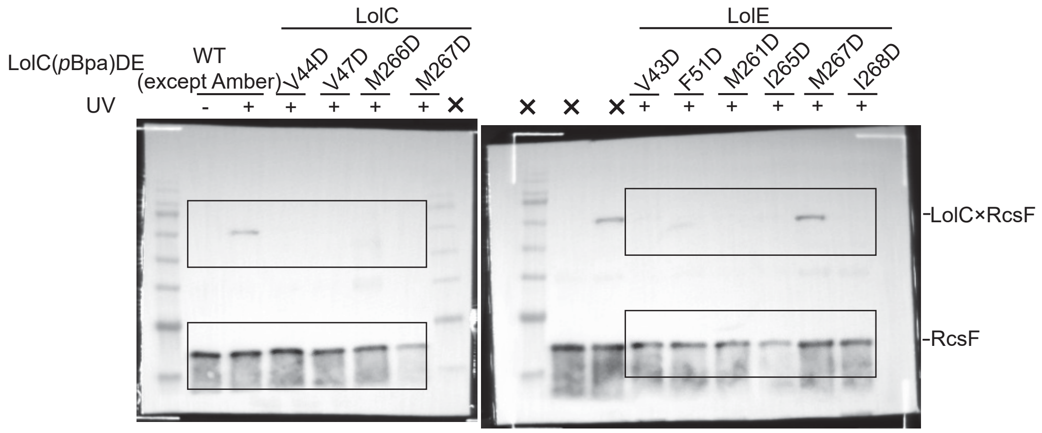

Image captured by ChemiScope3500 Mini (CLNX)

E

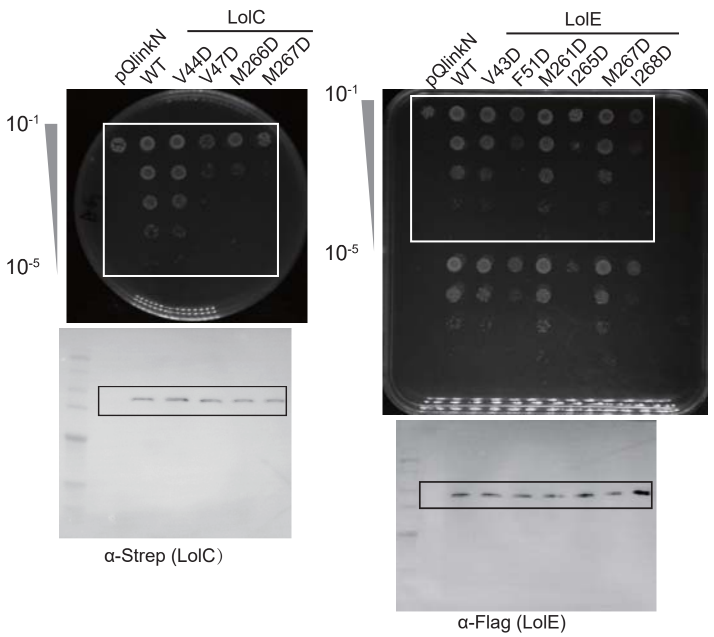

Image captured by CGenoSens and hemiScope3500 Mini (CLNX)

G

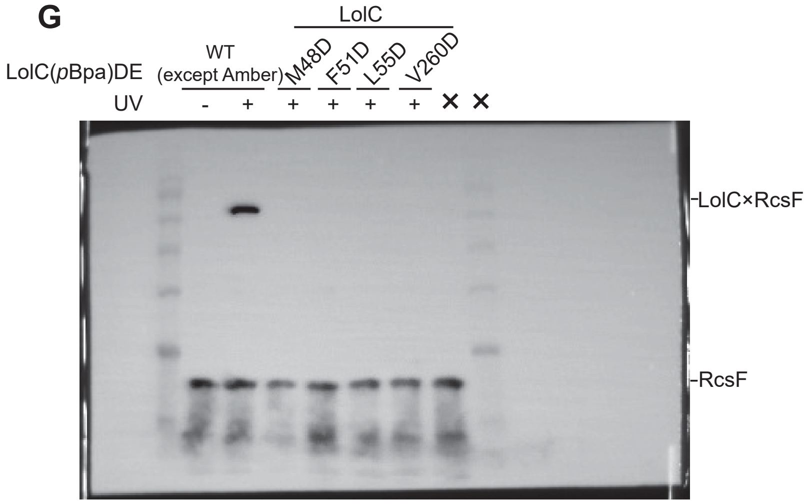

Image captured by ChemiScope3500 Mini (CLNX)

H

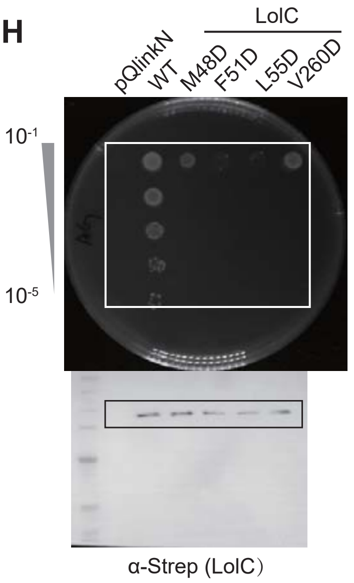

Image captured by CGenoSens and hemiScope3500 Mini (CLNX)

I

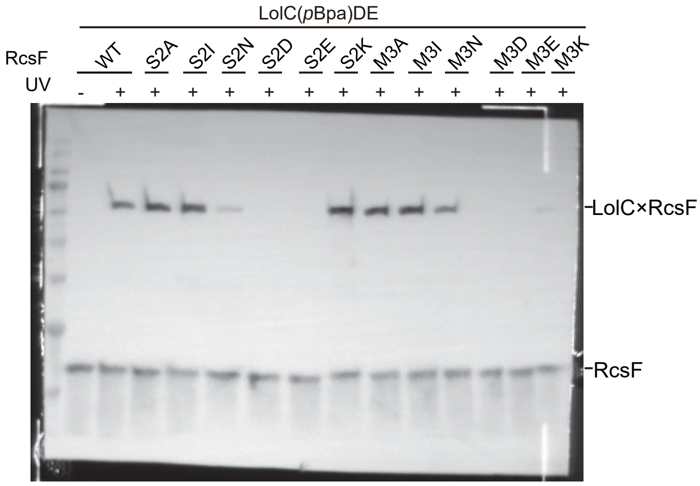

Image captured by ChemiScope3500 Mini (CLNX)

Fig 4. Functional importance of the negatively-charged residues in the V-shaped cavity

B

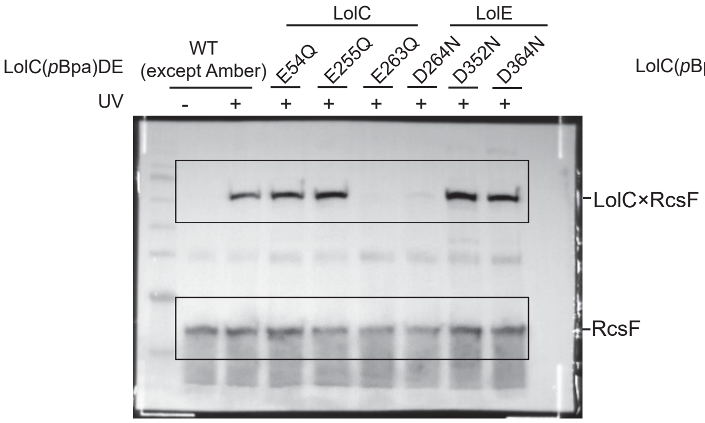

Image captured by ChemiScope3500 Mini (CLNX)

E

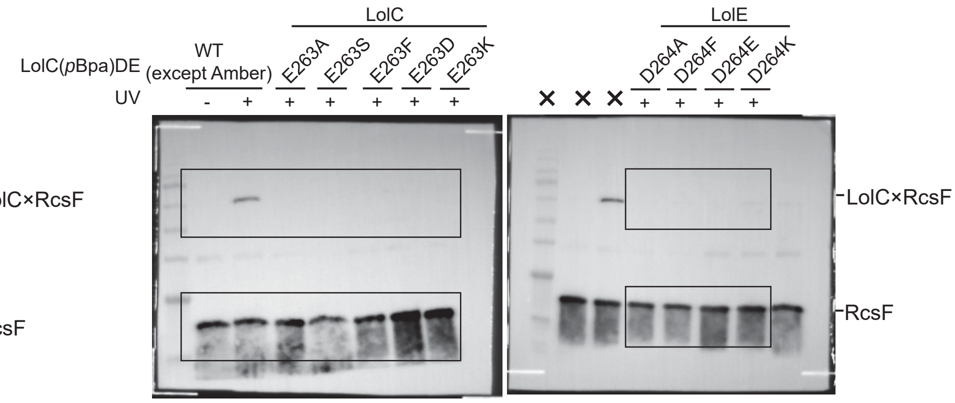

Image captured by ChemiScope3500 Mini (CLNX)

C

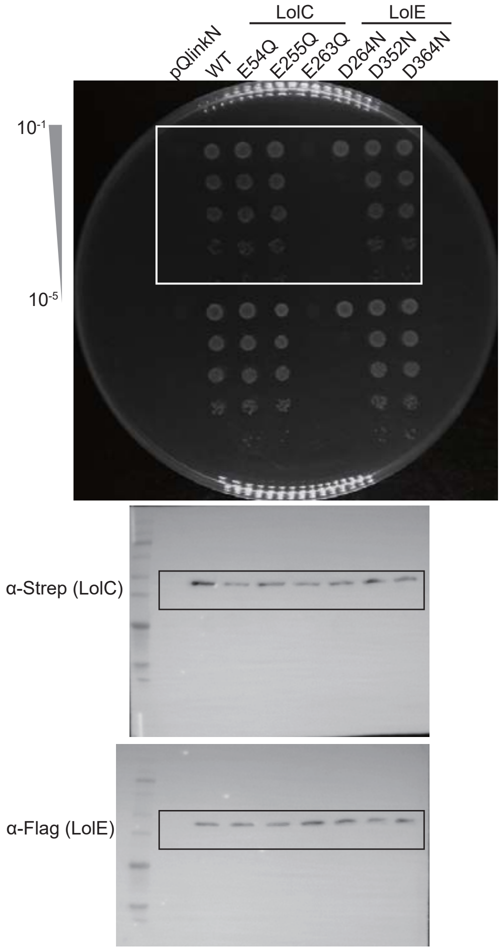

Image captured by GenoSens and ChemiScope3500 Mini (CLNX)

F

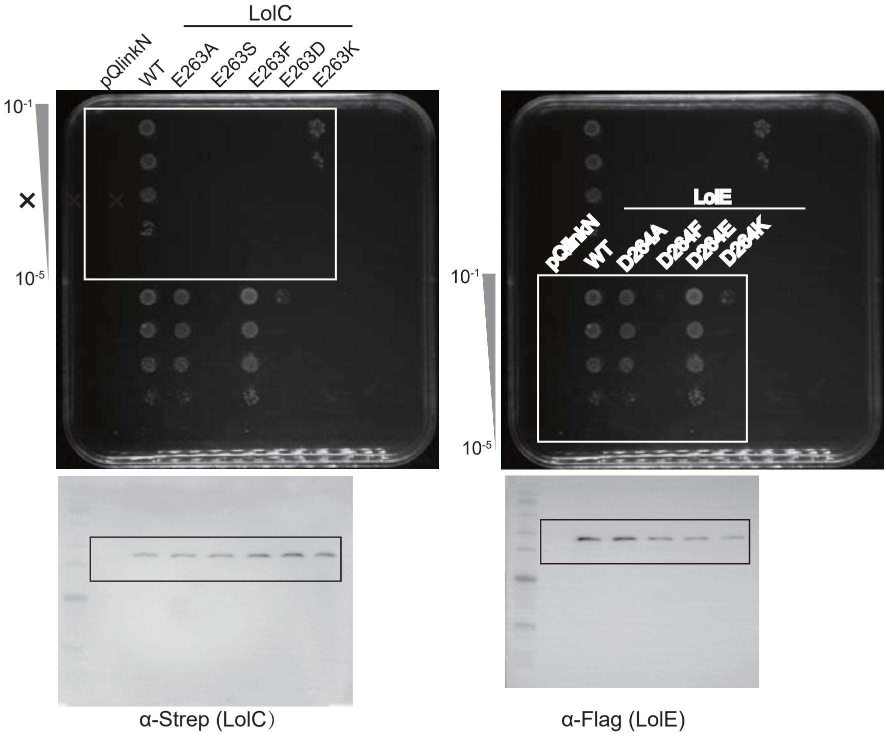

Image captured by GenoSens and ChemiScope3500 Mini (CLNX)

Fig 5. The U-loop maintains the configuration of the substrate-binding cavity

B

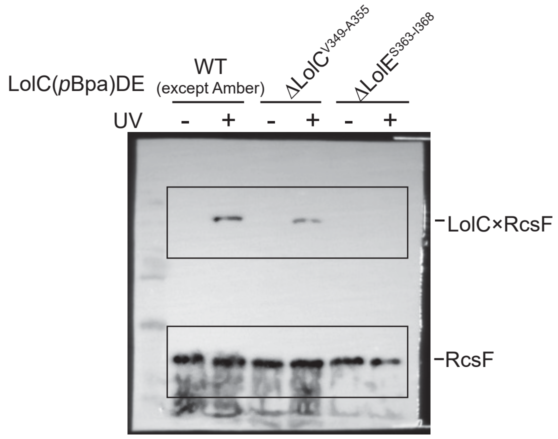

Image captured by ChemiScope3500 Mini (CLNX)

C

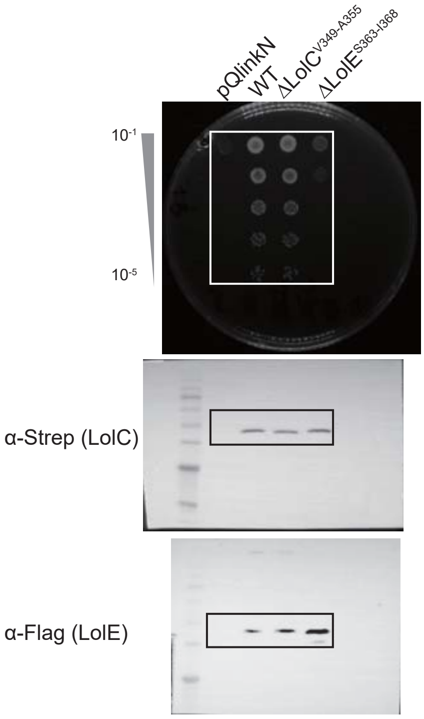

Image captured by GenoSens and ChemiScope3500 Mini (CLNX)

D

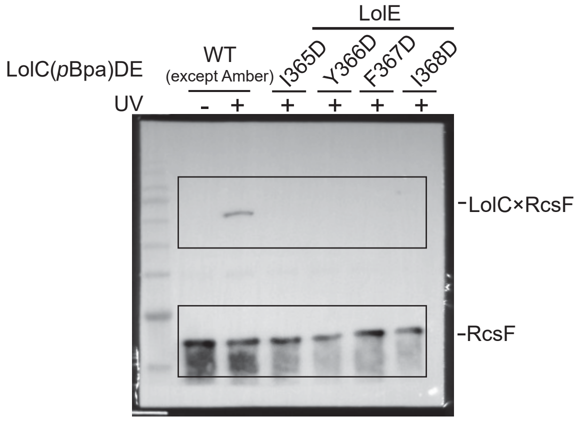

Image captured by ChemiScope3500 Mini (CLNX)

E

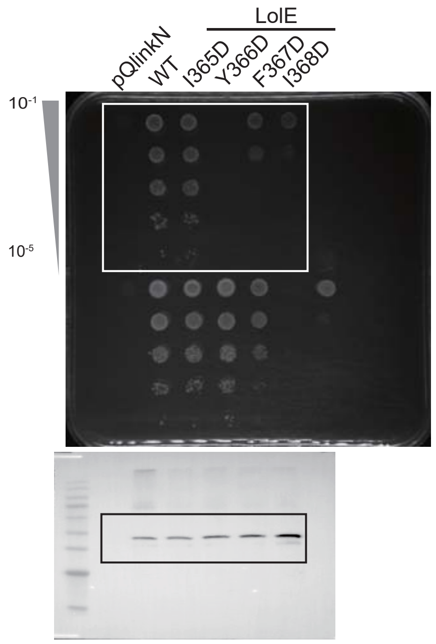

α-Flag (LolE)

Image captured by GenoSens and ChemiScope3500 Mini (CLNX)

Fig 6. A single path for lipoprotein entry into the V-shaped cavity and energy requirement for lipoprotein transfer to LolA

B

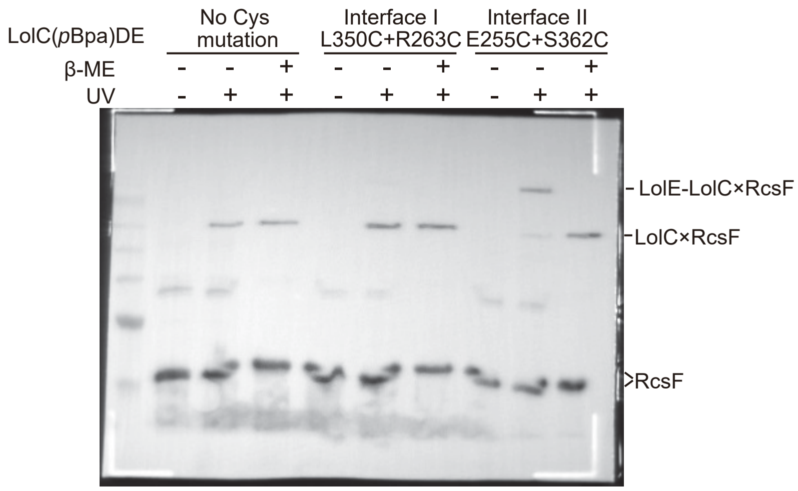

Image captured by ChemiScope3500 Mini (CLNX)

C

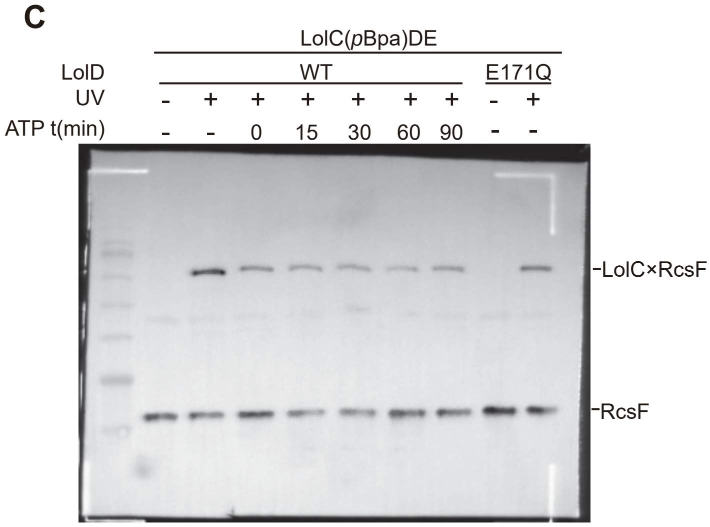

Image captured by ChemiScope3500 Mini (CLNX)

E

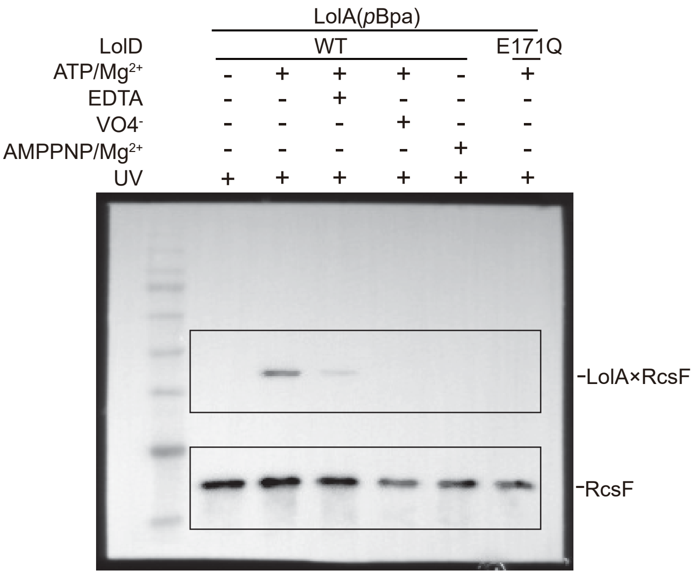

Image captured by ChemiScope3500 Mini (CLNX)

S1 Fig. Purification and reconstitution of LolCDE and RcsF-LolCDE in nanodisc

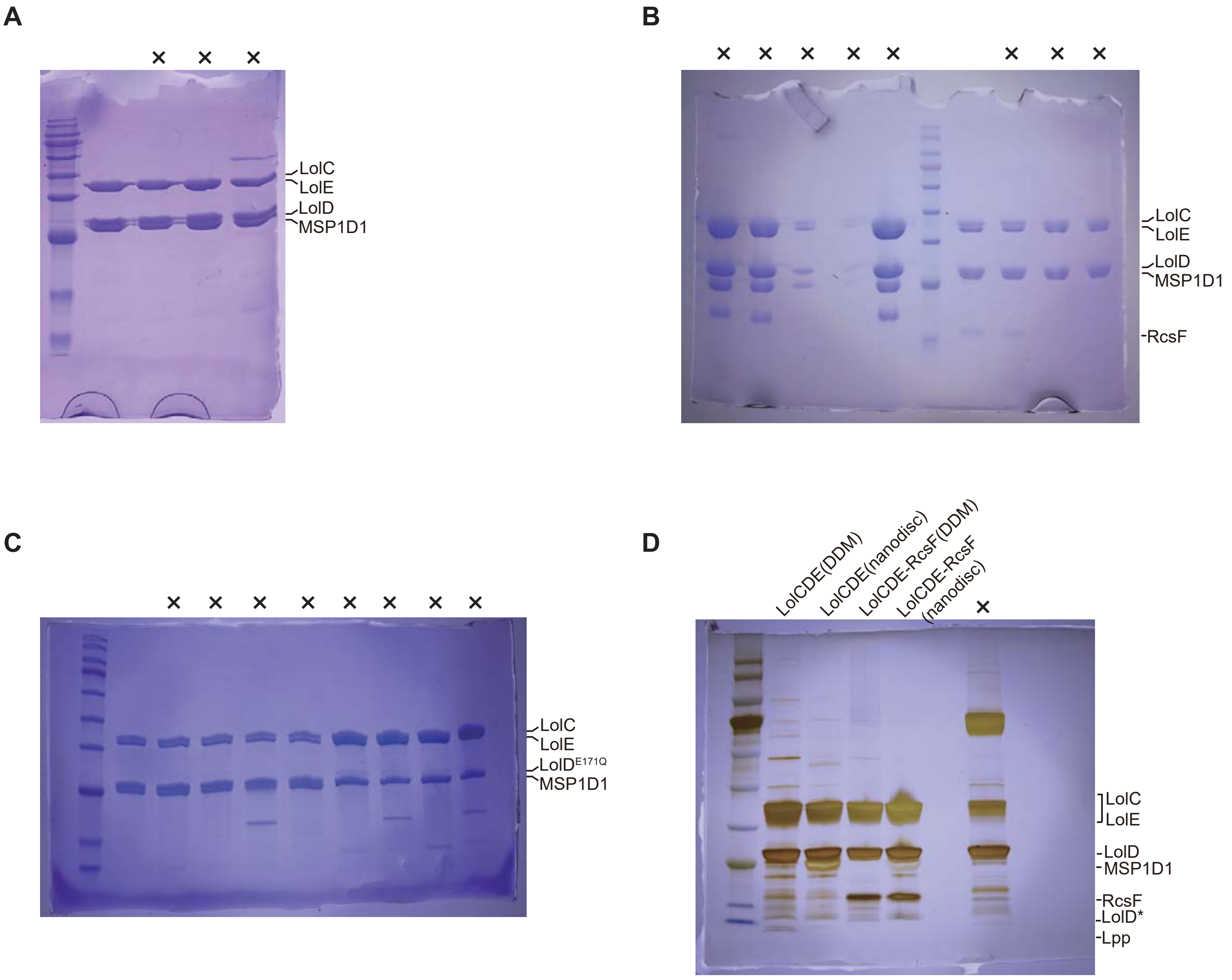

Image captured by camera

S11 Fig. The protein stability evaluation of the wildtype *lolCDE* and *lolCDE* mutant proteins by size exclusion chromattography

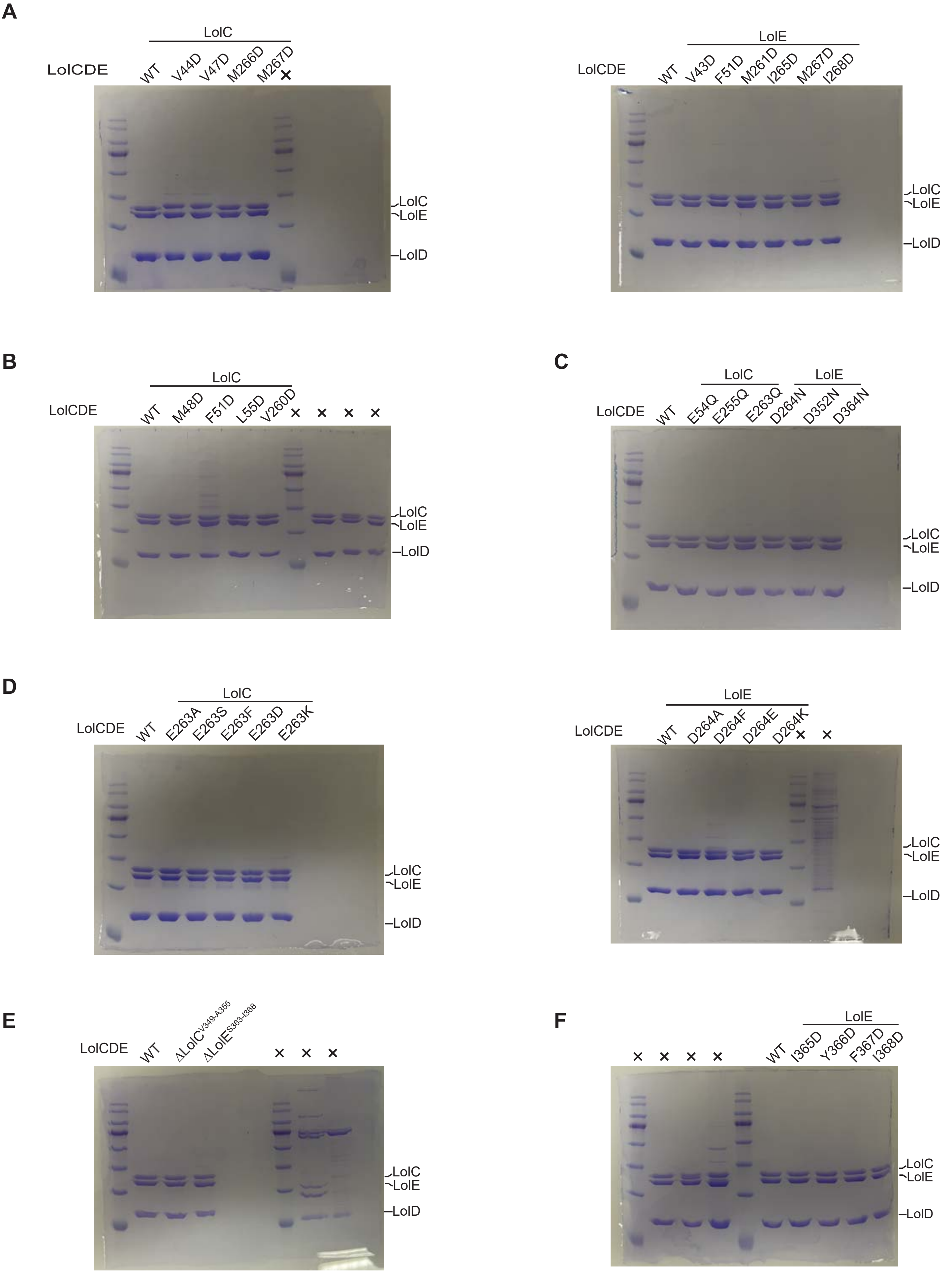

Image captured by camera

S12 Fig. Coomassie-stained SDS–PAGE gel analysis as loading control for the Western blots of photo-crosslinking assays in Figs. 3-5

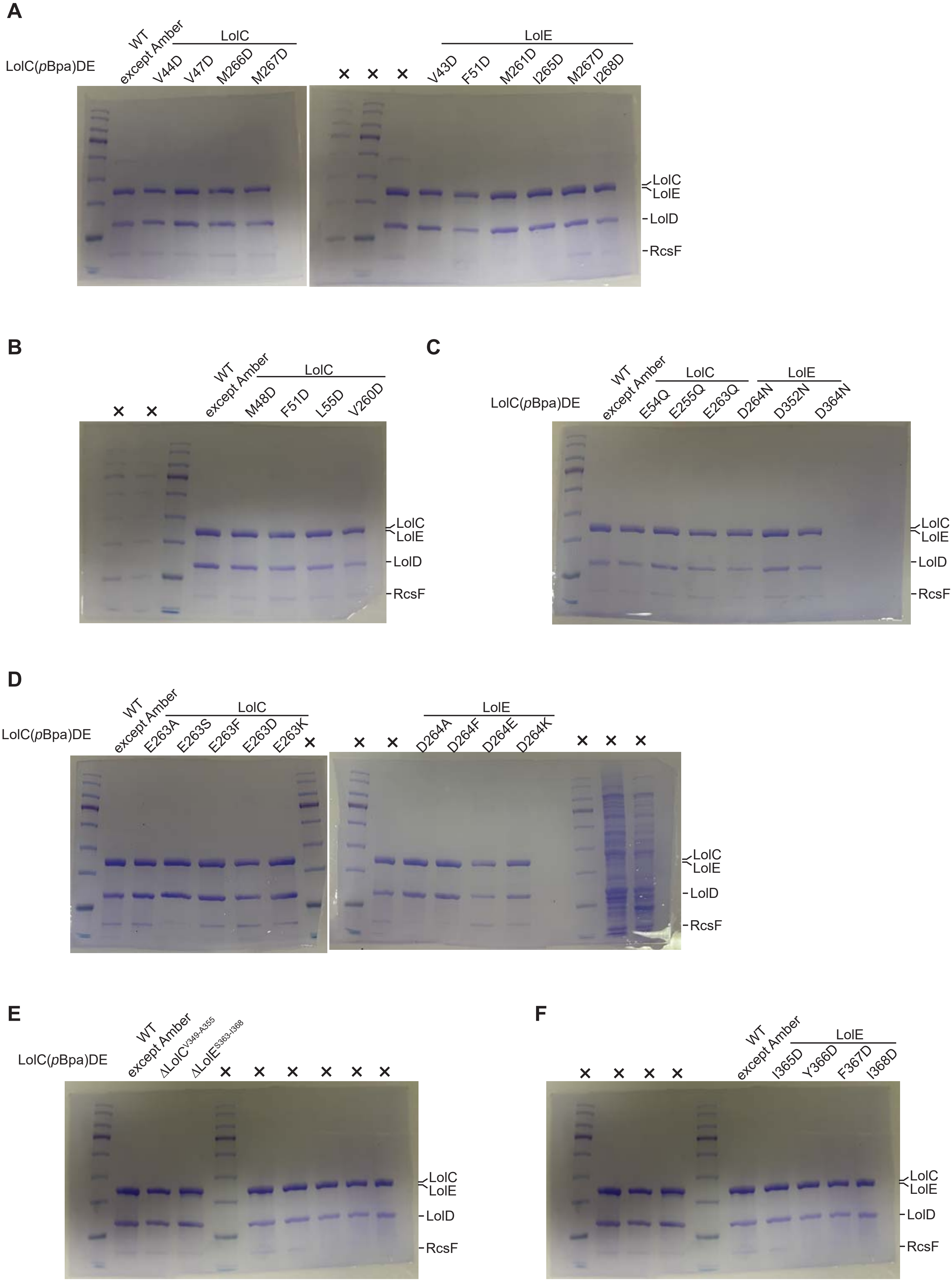

Image captured by camera

S13 Fig. Lipoprotein selectivity by LolCDE

D

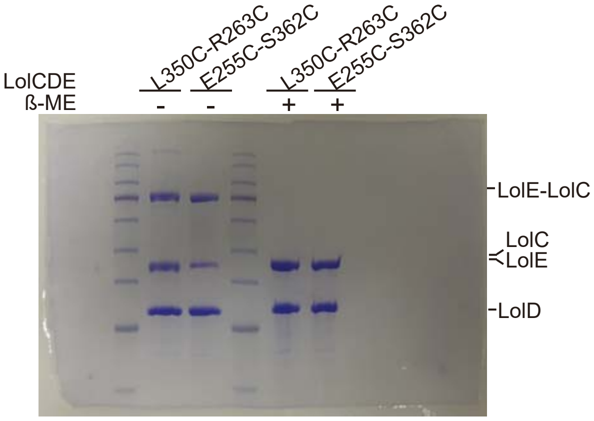

Image captured by camera

F

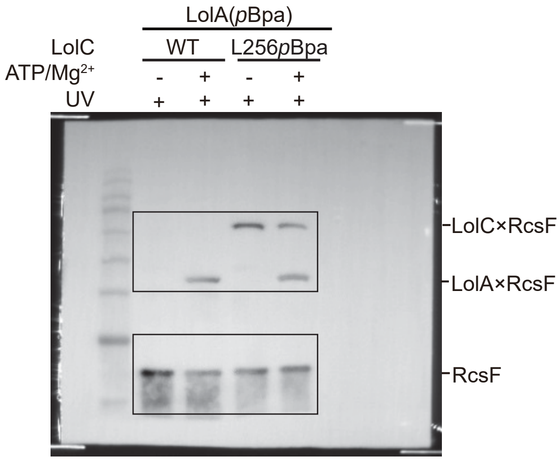

Image captured by ChemiScope3500 Mini (CLNX)
